# Regulation of Inhibitory Signaling at the Receptor and Cellular Level; Advances in Our Understanding of GABAergic Neurotransmission and the Mechanisms by Which It Is Disrupted in Epilepsy

**DOI:** 10.3389/fnsyn.2022.914374

**Published:** 2022-06-15

**Authors:** Allison E. Tipton, Shelley J. Russek

**Affiliations:** ^1^Graduate Program for Neuroscience, Boston University, Boston, MA, United States; ^2^Biomolecular Pharmacology Program, Boston University School of Medicine, Boston, MA, United States; ^3^Department of Pharmacology and Experimental Therapeutics, Boston University School of Medicine, Boston, MA, United States; ^4^Center for Systems Neuroscience, Boston University, Boston, MA, United States; ^5^Boston University MD/PhD Training Program, Boston, MA, United States

**Keywords:** GABAergic, inhibition, epilepsy, GABR transcription, GABA-A receptor, GABAAR trafficking

## Abstract

Inhibitory signaling in the brain organizes the neural circuits that orchestrate how living creatures interact with the world around them and how they build representations of objects and ideas. Without tight control at multiple points of cellular engagement, the brain’s inhibitory systems would run down and the ability to extract meaningful information from excitatory events would be lost leaving behind a system vulnerable to seizures and to cognitive decline. In this review, we will cover many of the salient features that have emerged regarding the dynamic regulation of inhibitory signaling seen through the lens of cell biology with an emphasis on the major building blocks, the ligand-gated ion channel receptors that are the first transduction point when the neurotransmitter GABA is released into the synapse. Epilepsy association will be used to indicate importance of key proteins and their pathways to brain function and to introduce novel areas for therapeutic intervention.

## Introduction

Epilepsy is a chronic neurological disorder characterized by repeated, unprovoked seizures. Seizure prevalence in those with epilepsy can range from one seizure every few months, to hundreds of seizures a day, the latter of which is usually seen in cases of genetic epilepsies (Epilepsy data and statistics CDC, 2020). Yet, even for those individuals who only experience seizures on rare occasions, the unpredictability of seizure occurrence often results in avoidance of social situations and a loss of independence that comes from a restriction in many of the abilities we take for granted, such as driving and even crossing the street alone.

Neurological disorders characterized as epilepsy can emerge throughout the lifespan, with cases of genetic epilepsy syndromes emerging in the first few years of life, childhood-restricted epilepsies, and new onset epilepsy in adulthood (Beghi, 2020). Frequently, adult-onset epilepsy stems from some type of insult to the brain, including traumatic brain injuries, strokes, or a single prolonged seizure [status epilepticus (SE)], and is commonly referred to as acquired epilepsy, of which temporal lobe epilepsy (TLE) is the most common (Téllez-Zenteno and Hernández-Ronquillo, 2012; Asadi-Pooya et al., 2017). At any given time, more than 1% of Americans are living with active epilepsy, and worldwide more than 50 million individuals have epilepsy (Epilepsy, 2022).

Though numerous classes of medications have come into use for epilepsy in the past several decades, nearly all of them come with a heavy load of side effects that may exacerbate symptoms co-morbid with epilepsy such as mood disorders and learning and memory difficulties (Keezer et al., 2016). Additionally, while anti-seizure medications provide seizure control for many patients, breakthrough seizures still occur, often with devastating effects. In addition, nearly one in three individuals with TLE cannot achieve seizure freedom with existing medications and often elect to undergo invasive surgery to remove portions of their temporal lobe in the hopes of eliminating the seizure loci (Asadi-Pooya et al., 2017). Perhaps more important than the unwanted side effects and lack of efficacy for many patients with epilepsy, all existing “epilepsy” medications are more correctly classified as anti-seizure drugs, as they do nothing to modify the disease, only treat the symptoms (Rogawski et al., 2016; Sills and Rogawski, 2020; Löscher and Klein, 2021).

For a long time, epilepsy has been viewed, at a most basic level, as a disorder of disrupted inhibition/excitation balance, favoring the latter and leading to repeated, synchronous firing not adequately constrained by inhibitory signaling (Fritschy, 2008; Hines et al., 2012). Initial support for this theory came from pharmacological studies, in which glutamate receptor agonists, such as kainic acid, or the toxin domic acid, were seen to be capable of inducing acute seizures (Ben-Ari et al., 1980; Sperk, 1994; Ramsdell, 2010). Additionally, early classes of anti-seizure drugs, and indeed most of those currently in use today, work by broadly decreasing excitation, or increasing inhibition, as is the case for barbiturates and benzodiazepines, both of which augment the function of type A γ-aminobutyric acid (GABA) receptors (GABA_A_Rs) (Griffin et al., 2013; Rogawski et al., 2016; Sills and Rogawski, 2020; Löscher and Klein, 2021). Further evidence in favor of a disruption in inhibitory/excitatory balance in epilepsy came from early studies using animal models of acquired epilepsy, and alterations in the levels of GABA_A_Rs seen in surgically resected or post-mortem tissue from patients with intractable epilepsy (Schwarzer et al., 1997; Tsunashima et al., 1997; Shumate et al., 1998; Houser and Esclapez, 2003; Pirker et al., 2003; Peng et al., 2004; Nishimura et al., 2005; Zhang et al., 2007).

Replication of these studies, along with more recent immunohistochemical datasets from surgically resected tissue of patients with intractable epilepsy, have produced inconsistent findings that often do not validate earlier reports (Schwarzer et al., 1997; Tsunashima et al., 1997; Shumate et al., 1998; Houser and Esclapez, 2003; Pirker et al., 2003; Peng et al., 2004; Nishimura et al., 2005; Zhang et al., 2007; Drexel et al., 2013; González et al., 2013, 2015; Stefanits et al., 2019; Sperk et al., 2021). Additionally, recent discoveries of mutations in epilepsy syndromes in ion channels that would be expected to promote increased inhibition, such as SCN1A and potassium channel gain of function mutations, along with upregulation of inhibition in models of absence epilepsy, suggest that E/I imbalance as a general predisposing factor for epilepsy may not be a sufficient descriptor of the condition itself (Miri et al., 2018; Shao et al., 2019; Owen et al., 2021).

Over the past 20 years there has been an expanded exploration into the basic mechanisms of inhibitory plasticity. A complex and dynamic signaling landscape has emerged that contains unique cell populations which deliver GABA into the synapse and a tightly controlled feedback system that regulates its receptors in discrete cellular compartments of specialized neuronal populations. In this review, we will examine various mechanisms that have been shown to alter inhibitory signaling, with a special focus on GABA_A_Rs and their efficacy as the brain’s major inhibitory sensor. We will explore regulation of (1) GABA receptor trafficking, stabilization, and localization, (2) GABA_A_R subunit composition (with associated changes in channel properties), (3) inhibitory synapse formation and elimination, and identification of (4) vulnerable GABAergic neuronal populations. Additionally, we will provide examples where a particular nexus of control is known, or proposed, to be dysregulated with epilepsy, either in animal models or in tissue samples collected from human epilepsy patients. Finally, we will discuss how the proliferation of single cell technologies and genetic disease models may allow for a more refined understanding of inhibitory brain dynamics that impact seizure disorders and how this may inform the development of more targeted therapies.

## GABA-A Receptor Trafficking, Stabilization, and Localization

For GABA_A_Rs to exert an inhibitory effect in neurotransmission, they must be located within the plasma membrane, either at the synapse (synaptic) or away from the synapse (extrasynaptic). Given the need for fine-tuned control of inhibitory signaling, neurons possess numerous mechanisms that they use to dynamically regulate GABA_A_Rs at the cell surface (Luscher et al., 2011). This includes the initial formation of the pentameric receptor, that requires specific subsets of receptor subunits to participate in an assembly process within the endoplasmic reticulum (ER) or to be tagged for turnover in a ubiquitin- dependent manner. Once GABA_A_Rs are assembled, they next travel to the Golgi apparatus where specific subunits can undergo modifications, such as palmitoylation, before being packaged into vesicles for trafficking to the plasma membrane. Initially, GABA_A_Rs are inserted at extrasynaptic sites, from which they can laterally diffuse to the synapse (Jacob et al., 2005). Following insertion into the plasma membrane, they may be stabilized extrasynaptically and/or undergo dynamic lateral diffusion between synaptic and extrasynaptic compartments, influenced by interactions with other proteins, as well as posttranslational modifications (Vithlani et al., 2011). Additionally, GABA_A_Rs undergo dynamic endocytosis and endocytic cycling, with additional interacting proteins, such as huntingtin-associated protein 1 (HAP1), and the phosphorylation status of a particular subunit, determining whether they are recycled back to the cell surface membrane, or targeted to the proteasome for degradation (Lorenz-Guertin et al., 2018).

Broadly speaking, cellular processes that regulate the presence of GABA_A_Rs at the plasma membrane can be divided into GABA receptor trafficking, localization, and stabilization; each is discussed in turn in the following sections. [For an in-depth review of proteins and processes, see Moss et al. (2008), Luscher et al. (2011), Mele et al. (2019)].

### Regulation of GABA-A Receptor Trafficking

#### Trafficking of Receptors to the Plasma Membrane

**FIGURE 1:**
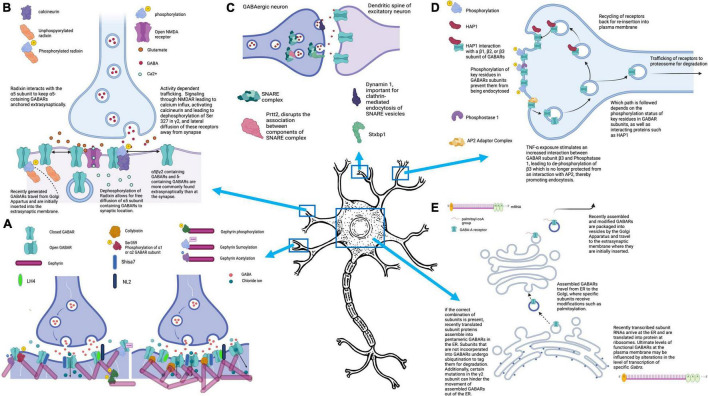
Mechanisms that control GABA_A_Rs-mediated inhibitory signaling. **(A)** The number of GABA_A_Rs clustered at the synapse can directly impact the strength of the inhibitory current generated by GABA release into the synaptic cleft. GABA_A_R clustering is promoted by interactions of GABA_A_Rs with binding partners including gephyrin and collybistin. Several posttranslational modifications, including phosphorylation of Ser-359 at α1 or α2, or sumoylation, phosphorylation, or acetylation of Gephyrin, impair GABA_A_R clustering, resulting in fewer synaptic GABA_A_Rs. Mutations of collybistin have been associated with genetic epilepsies. **(B)** GABA_A_Rs are located both synaptically and extrasynaptically. At the synapse, they modulate fast inhibition (phasic), and extrasynaptic GABA_A_Rs mediate tonic inhibition. All GABA_A_Rs are initially inserted into the plasma membrane extrasynaptically, but can then laterally diffuse to the synapse. Under normal conditions, Radixin serves to keep α5-containing GABA_A_Rs at extrasynaptic sites, but this association can be disrupted, allowing for diffusion of α5-containing GABA_A_Rs to the synapse. Diffusion of GABA_A_Rs away from the synapse can be triggered by activity dependent trafficking, in which calcium influx through activated NMDARs leads to activation of phosphatase calcineurin and subsequent desphosphorylation of the γ2 subunit at Ser-327. **(C)** The efficacy of GABAergic synapses can be influenced by pre-synaptic factors, including proteins that play a part in NT exocytosis. Several of the proteins that participate in this process have been found to be mutated in epilepsy syndromes, including Stxbp1, Prrt2, and Dnm1. **(D)** There is dynamic movement of GABA_A_Rs into and out of the plasma membrane, with regular endocytosis of receptors. The fate of these endocytosed receptors depends on the phosphorylation status of the subunits, as well as interaction with accessory proteins, like HAP1, which facilitates movement of endocytosed GABA_A_Rs back to the plasma membrane rather than targeting them for proteosomal degradation. **(E)** GABA_A_Rs are initially brought together into their pentameric structure in the endoplasmic reticulum. Within the ER, specific subunits must be present or else all subunits will be ubiquinated. If a GABA_A_R is successfully formed in the ER, it next travels to the Golgi Apparatus where it undergoes post-translational modifications including palmitoylation. After passage through the Golgi, GABA_A_Rs are packaged into vesicles and travel to the cell membrane for insertion at extrasynaptic sites. Figure generated using BioRender.com.

Trafficking of GABA_A_Rs begins with the assembly of the receptor subunits into a pentamer in the ER, followed by their movement to the Golgi apparatus, where the pentameric receptor undergoes additional modifications before arriving at the plasma membrane (Figure 1E). The γ2 subunit, which is found in up to 90% of GABA_A_Rs in the brain, plays an important role in facilitating the movement of newly assembled GABA_A_Rs out of the ER, and varying mutations in this subunit have been discovered in cases of genetic epilepsies (Huang et al., 2014; Kang, 2017; Lorenz-Guertin et al., 2018). In fact, not only do mutations in GABA_A_R subunits result in a receptor with altered functionality, but the mutant protein can suppress expression and trafficking of the wildtype protein leading to a failure of γ2-containing GABA_A_Rs to exit the endoplasmic reticulum (Kang et al., 2013; Kang, 2017). Interestingly, a recent paper by Yuan and colleagues identified Cleft lip and palate associated transmembrane protein 1 (CLPTM1) as a negative regulator of the movement of GABA_A_Rs to the membrane, instead trapping them in the ER and/or Golgi apparatus leading to decreased strength of the resultant inhibitory synapses (Ge et al., 2018). A tandem affinity purification assay was used to isolate YFP-tagged γ2 from transgenic mice and mass spectrometry was used to identify proteins in complex with γ2-containing GABA_A_Rs. This assay identified 39 potential candidates, aside from those already known to interact with γ2 that may play a role in modulating GABA_A_R density at synapses. Three of these 39 interactions were validated via *in vitro* studies in transfected HEK293 cells, including CLPTM1, Integral membrane protein 2C (Itm2c), and Golgi glycoprotein 1 (Glg1). However, only the presence of CLPTM1 altered GABAergic currents, leading to decreased amplitude of GABA_A_R mediated currents, but having no effect on the frequency of currents. Importantly, by preventing the progression of GABA_A_Rs out of the ER, and to the plasma membrane, CLPTM1 modulates both tonic and phasic inhibition.

Additional regulation of the trafficking of GABA_A_Rs to the plasma membrane comes from proteins that play a role in promoting re-insertion of receptors that have undergone endocytosis (Figure 1D). One such protein is HAP1 that, under normal conditions, promotes the trafficking of endocytosed GABA_A_Rs back to the plasma membrane rather than to the lysosome for endocytosis degradation (Kittler et al., 2004). Kittler and colleagues determined that direct interactions of HAP1 with endocytosed GABA_A_Rs protects them from degradation (Kittler et al., 2004). In fact, they demonstrate that HAP1 is a label used by cells to prevent GABA_A_R turnover and instead, promote receptor recycling back to the plasma membrane of synapses. Importantly, this leads to greater numbers of cell surface GABA_A_Rs without a compensatory increase in endocytosis, leading to an increased magnitude of miniature inhibitory postsynaptic currents (mIPSC’s). This important study also highlighted the specificity of the HAP1- GABA_A_R subunit interaction which occurs with β1, β2, or β3 subunits. Given that all GABA_A_Rs will have one of these β subunit subtypes, HAP1 could have an important role in the receptor recycling of most GABA_A_Rs in the nervous system (Kittler et al., 2004).

#### Trafficking of Receptors Away From the Plasma Membrane (Endocytosis)

Research has demonstrated that there is a continuous cycling of GABA_A_Rs into and out of the plasma membrane, such that any one receptor does not remain at the cell surface for long (Thomas et al., 2005). However, under steady state conditions, there is an equal rate of insertion and endocytosis and thus no net change in receptor levels at the plasma membrane. Trafficking of receptors away from the membrane; however, can provide a means to dynamically modulate inhibitory signaling when needed by disturbing this equilibrium.

Interestingly, TNF-alpha causes increased association between β3 and protein phosphatase 1 (PP1), leading to de-phosphorylation of key residues on the subunit. Due to this dephosphorylation event, the β3 is no longer protected from association with endocytic machinery, mainly the adaptor protein AP2 (Figure 1D). Ultimately, this leads to increased endocytosis of β3-containing GABA_A_Rs and decreased inhibitory synaptic strength. In fact, Pribiag and colleagues found that TNF-alpha exposure impaired both the magnitude and frequency of inhibitory postsynaptic currents (IPSC’s) (Pribiag and Stellwagen, 2013).

One important setting in which GABA_A_R endocytosis plays a critical role is in activity dependent trafficking, both under physiologic and pathologic conditions (Figure 1B). Perhaps the most significant physiological condition under which GABA_A_Rs are trafficked away from the synaptic membrane is in response to excitatory signaling through N-methyl-D-aspartate receptors (NMDARs) (Muir et al., 2010; Eckel et al., 2015). Part of the role of this trafficking is to allow for excitatory synapses to be strengthened in the setting of learning, and its potential cellular correlate long-term potentiation, which would not be possible if a targeted increase in excitatory glutamatergic signaling was met with a reciprocal increase in GABAergic. And yet, despite these early findings of induction of GABA_A_R dispersion due to NMDAR-mediated excitatory signaling, additional investigations have uncovered a potential role for NMDAR activation in inhibitory long-term potentiation (iLTP), and even inhibitory synaptogenesis (Marsden et al., 2007; Petrini et al., 2014; Gu et al., 2016; Chiu et al., 2018; Wu et al., 2021a).

An early paper demonstrated that the same mechanism that drives the NMDAR activation of AMPA receptor endocytosis, in the context of LTD, regulates the presence of GABA_A_Rs at the cell surface. This receptor-dependent potentiation of inhibitory synaptic plasticity relied on NMDAR-mediated calcium influx, and the activation of Calcium/Calmodulin-dependent protein kinase II (CAMKII). The investigators also identified a necessary role for N-Ethylmaleimide sensitive factor (NSF), GABA Type A receptor-associated protein (GABARAP), and Glutamate receptor interacting protein (GRIP) in the insertion of new synaptic GABA_A_Rs in response to NMDAR activation (Marsden et al., 2007). Several papers have recently expanded on this earlier work, demonstrating an essential role for NMDARs in the potentiation of both phasic and tonic inhibition.

Using optogenetics to stimulate GABAergic neurons *in vivo* in the mouse medial prefrontal cortex (mPFC), Chiu and colleagues identified a form of inhibitory potentiation at dendrites that occurs in response to calcium influx through NMDARs (Chiu et al., 2018). This iLTP is not found in interneurons expressing either Vasoactive intestinal peptide (VIP) or Parvalbumen (PV). Only Somatostatin (Sst) positive interneurons express this physiological response which is dependent on calcium induced activation of CAMKIIa and GABA_A_Rs containing the β2 subunit. Moreover, through use of various pharmacological inhibitors, they demonstrate that activation of NMDARs, specifically Glutamate 2B (GluN2B), was required, rather than simply CAMKIIa activation from any calcium source. A role for CAMKII in iLTP was also demonstrated in a 2014 report that focused on mechanisms leading to increased clustering and the stabilization of GABA_A_Rs at synapses, again in the setting of iLTP. The investigators found that increased clustering of GABA_A_Rs at synapses relies on CAMKII-mediated phosphorylation of Ser383 in the β3 subunit of GABA_A_Rs, leading to recruitment of gephyrin from extrasynaptic sites to synaptic. Here, gephyrin mediates not only the increased recruitment of GABA_A_Rs but their stabilization at synapses, playing an essential role in the long-term potentiation of inhibition (Petrini et al., 2014).

Studies from Wu, Castellano et al. specifically show that GluN2B and GluN2A-containing NMDARs reciprocally control insertion of extrasynaptic GABA_A_Rs, thus mediating tonic inhibitory currents. Interestingly, GluN2A-containing NMDA receptors are needed for tonic long-term potentiation in adaptation to prolonged alterations in neuronal activity. The impact of NMDAR subunit composition varied across development into adulthood. In immature neurons, signaling through GluN2B-containing NMDARs directly regulates the strength of tonic inhibition, whereas manipulation of those receptors with GluN2A was without effect. In contrast, in mature neurons, both GluN2B- and GluN2A containing–NMDARs serve to regulate the strength of tonic inhibition through promotion or blockade of endocytosis of α5 containing extrasynaptic GABA_A_Rs, respectively (Wu et al., 2021a).

In contrast, in epilepsy models where there is excessive excitation, activity-dependent trafficking of GABA_A_Rs can serve to make runaway excitation worse. Under normal conditions, increased neuronal activity driven by NMDA receptors leads to a calcium influx. This influx activates the phosphatase calcineurin, which then dephosphorylates Serine 327 on the GABA_A_R γ2 subunit, disrupting one of the stabilizing factors that normally keeps γ2-containing receptors at the synapse (Muir et al., 2010). In the absence of Serine 327 γ2 phosphorylation, GABA_A_Rs are free to diffuse laterally, ultimately resulting in fewer GABA_A_Rs at synapses (Figure 1B). In fact, additional studies have demonstrated a role for calcineurin in the alteration of GABAergic signaling efficacy, specifically in the setting of seizures (Eckel et al., 2015). Eckel and colleagues, in an *in vitro* model of SE, found that SE resulted in the downregulation of GABA_A_Rs at 20 and 60 minutes after induction of SE, an effect brought about by calcineurin (which subsequent studies have demonstrated alters a key phosphorylation site on the GABA_A_R subunit α2 so that it is no longer stabilized at the synapse) (Muir et al., 2010; Eckel et al., 2015; Nicholson et al., 2018).

Likewise, *in vivo* studies in the lithium chloride-pilocarpine SE model report that phosphatase inhibitors prevent the activity-dependent trafficking of γ2 subunit-containing GABA_A_Rs away from the synapse, suggesting that indeed, calcineurin-dependent dephosphorylation of γ2 promotes dispersal of γ2-containing GABA_A_Rs with increased neuronal activity (Joshi et al., 2015). Importantly, a similar mechanism involving calcineurin activation, downstream of a phospholipase C delta (PLC-δ)-mediated increase in intracellular calcium, has been shown to contribute to benzodiazepine pharmacoresistance in the treatment of SE in patients with long-term benzodiazepine treatment (Nicholson et al., 2018).

### Regulation of GABA(A) Stabilization

While there is always an exchange of GABA_A_Rs into and out of the plasma membrane under baseline conditions, the interaction of specific GABA_A_R subunits with selective proteins can influence the duration of time they spend at the membrane, and hence their stabilization.

The most important class of proteins responsible for the stabilization of GABA_A_R populations at the membrane are scaffolding proteins which help tether receptors in specialized units (Figure 1A). Gephyrin is the most widely known scaffolding protein in association with inhibitory synapses, and loss or downregulation of gephyrin impairs clustering of GABA_A_Rs at the synapse (Essrich et al., 1998; Kneussel et al., 1999, 2001; Lévi et al., 2004). Importantly, gephyrin can be regulated not just at the transcriptional level, but through its various post-translational modifications or its interacting partners that impact its association with GABA_A_Rs (Mukherjee et al., 2011, 2017; Tyagarajan et al., 2011, 2013; Wu et al., 2011; Kalbouneh et al., 2014; Flores et al., 2015; Ghosh et al., 2016). For example, phosphorylation of key residues on several of the receptor subunits, including Ser-359 of α2 and α1, impair the ability of these subunits and the receptors containing them to interact with gephyrin (Mukherjee et al., 2011; Nakamura et al., 2020). Gephyrin itself undergoes several key forms of post-translational modification, including phosphorylation, SUMOylation and acetylation (Tyagarajan et al., 2011, 2013; Ghosh et al., 2016). Interestingly, it seems that SUMOylation of residues within gephyrin impair its function as a scaffold for GABA_A_Rs and cause a resultant decrease in GABA_A_R clustering and magnitude of IPSC’s (Ghosh et al., 2016). Additionally, both acetylation and phosphorylation at key residues examined in this study resulted in differing impacts on the size and number of gephyrin clusters, with diminished gephyrin clustering resulting from both modifications. In fact, activity-dependent dispersion of GABA_A_Rs in part relies on phosphorylation of gephyrin and the resulting disruption of the clustering effects that the gephyrin scaffold provides (Flores et al., 2015).

Various intracellular pathways may alter the phosphorylation state of gephyrin, including mitogen-activated protein kinase (MAPK), glycogen synthase kinase 3 Beta (GSK3B), and cyclin-dependent kinase 5 (CDK5). Many of the exogenous factors that disrupt gephyrin clustering likely act through these pathways, including estradiol/estrogen and brain-derived neurotrophic factor (BDNF) (Tyagarajan et al., 2011, 2013; Wu et al., 2011; Kalbouneh et al., 2014; Flores et al., 2015; Mukherjee et al., 2017; Brady et al., 2018). Given the critical role of gephyrin in scaffolding at most inhibitory synapses, it is not unsurprising that disruptions to gephyrin function or structure are associated with epilepsy (Gonzalez, 2013; González et al., 2013, 2015; González, 2019; Garcia et al., 2021). For example, calpain, a key protease which gets activated during epileptogenesis, leads to subsequent cleavage of gephyrin, thus disrupting the clustering of GABA_A_Rs and the efficacy of inhibitory synapses (González, 2019). In a recent paper utilizing an oxygen and glucose deprivation model of excitotoxicity, the authors showed that at least two separate signaling pathways play a role in augmenting fast inhibitory signaling following an excitotoxic insult; one, which leads to disruption of GABA_A_R clustering at synapses, and is dependent on calcineurin, and the second, a pathway that relies on calpain and disrupts gephyrin assembly (Garcia et al., 2021).

It is now known that gephyrin is not the only protein that scaffolds GABA_A_Rs at the synapse, as gephyrin-independent clustering of γ2-containing GABA_A_Rs has been identified. In a search for potential auxiliary subunits associated with GABA_A_Rs, like those previously seen and defined at excitatory synapses, lipoma HMGIC fusion partner-like 3 and 4 [referred to as the GABA_A_R regulatory LFRPL (GARLH family proteins that contain LH3- and LH4-like protein)] were identified as putative auxiliary GABA_A_R subunits that act as alternative scaffolding molecules at GABAergic synapses (Yamasaki et al., 2017). In the absence of LHFPL4, fewer inhibitory synapses develop in a neuronal culture assay, and mice lacking LHFPL4 in cerebellar granule cells demonstrate enhanced susceptibility to seizures as well as disrupted motor behavior. Additionally, collybistin scaffolds GABA_A_Rs to the membrane through its binding to both GABA_A_R subunits and gephyrin itself (Kins et al., 2000; Papadopoulos et al., 2008). Mutations in collybistin have been directly implicated in genetic causes of epilepsy (Shimojima et al., 2011).

Other classes of proteins are also essential to the tethering or maintenance of GABA_A_Rs at the plasma membrane. One such protein, neuroligin 2 (NLGN2), serves to stabilize GABA_A_Rs at synapses by interacting with the γ2 subunit, in association with the GARLH protein LH4-like (see above) that helps to stabilize γ2-containing GABA_A_Rs at the synapse (Varoqueaux et al., 2006; Zhang et al., 2015; Yamasaki et al., 2017). In fact, LH4-like protein may even be important in the function of gephyrin with GABA_A_Rs, as its knockout (KO) leads to a decrease in gephyrin clustering (Davenport et al., 2017; Yamasaki et al., 2017). More functional diversity comes from the contribution of S-SCAM, a cell adhesion molecule that interacts with NLGN2, immunoglobulin superfamily member 9B (IGSF9B), and β-dystroglycan in its role as a GABA_A_R scaffolding protein (Shin et al., 2020). Interestingly, researchers have demonstrated that the level of S-SCAM must be controlled within a tight range of expression, as both up- and downregulation attenuates the function of GABAergic synapses. Importantly, deletion of one copy of the S-SCAM gene leads to infantile spasms, one of the most severe forms of epileptic encephalopathy (Marshall et al., 2008; Kang, 2017; Shin et al., 2020).

Mutations in protocadherin 19 (PCDH19), a protein involved in stabilization of extrasynaptic GABA_A_Rs that contribute to tonic inhibition, are also associated with another severe form of epilepsy: epileptic encephalopathy, early infantile 9 (Dibbens et al., 2008; Smith et al., 2018; Kolc et al., 2019; Serratto et al., 2020). PCDH19 binds directly to GABA_A_Rs and plays a role in GABA_A_R recycling and miniature IPSC’s. In a study by Serratto and colleagues, normal PCDH19 expression was found to be important for baseline tonic inhibition, while its downregulation leads to reduced GABA_A_R mediated tonic currents (Serratto et al., 2020).

### Regulation of GABA(A) Receptor Localization

The role played by GABA_A_Rs in brain inhibition is influenced not only by their presence at the plasma membrane, as opposed to the abundant intracellular pool of GABA_A_R subunits, but critically whether they are located synaptically or extrasynaptically. Most importantly, there is a ready exchange between synaptic and extrasynaptic sites for receptors via lateral diffusion (Thomas et al., 2005; Bogdanov et al., 2006; Bannai et al., 2009; Hannan et al., 2020).

Localization of GABA_A_Rs is in part influenced by their subunit composition, with certain receptor subtypes primarily localizing in one compartment vs the other (Nusser et al., 1996; Caraiscos et al., 2004; Sun et al., 2004; Chandra et al., 2006; Glykys et al., 2008). For example, historical research has documented the primary subunit composition of GABA_A_Rs in the hippocampus and shown that in the dentate gyrus, synaptic receptors tend to be α1βγ2 or α2βγ2 receptors, while extrasynaptic are α4βδ-containing receptors (Nusser et al., 1996; Sun et al., 2004; Chandra et al., 2006). In contrast, in the CA1 region of the hippocampus, extrasynaptic receptors are found to be primarily α5βγ2-containing receptors, illustrating heterogeneity of GABA_A_Rs even within a single region of the brain (Caraiscos et al., 2004; Glykys et al., 2008). Despite the existence of specific subunit assemblies for synaptic and extrasynaptic receptors, these are not hard and fast rules. A recent study from Saad et al. demonstrated that although GABA_A_Rs containing specific subunits are more likely to be found synaptically or extrasynaptically, all the receptor subunits under study (including α2, α4, α5, and δ) could be found at the synapse (as opposed to extrasynaptically). However, certain receptor subunit combinations were found more often at the synapse than others(Hannan et al., 2020).

Interestingly, the dwell time for α2 and α4 subunit-containing receptors at synapses was longer than those containing α5 or δ, which could help explain why δ and α5 are more likely to be found extrasynaptically. Under conditions with greater need for inhibitory signaling, receptor activation can result in increased lateral mobility of extrasynaptic versus synaptic receptors, such that extrasynaptic receptors can diffuse to the synapse and lead to increased hyperpolarization/amplitude of fast synaptic signaling (Hannan et al., 2020). However, instances have been reported where such recruitment decreases rather than increases brain inhibition, as extrasynaptic receptors have a higher affinity for GABA and can desensitize rapidly in this new environment (Lagrange et al., 2007).

In addition to subunit composition, specific interacting partners of GABA_A_Rs impact their localization. There are numerous proteins that play a role in the localization of GABA_A_Rs specifically to synaptic locations. Signaling via Noga-A, one of the most potent inhibitors of neurite outgrowth in the central nervous system, helps increase localization of GABA_A_Rs to the synapse by preventing the lateral diffusion of GABA_A_Rs in the compartment (Fricke et al., 2019). Also important for synaptic localization of GABA_A_Rs, is protrudin that was first associated with GABAergic signaling due to studies showing an association between a mutation in its gene and hereditary spastic paraplegia (Lu et al., 2019). In their 2019 paper, Lu et al. (2019) confirmed the relevance of protrudin to epilepsy by first examining its expression levels in tissue from human TLE and animal models where they found *downregulation* was *detrimental*. They then went on to overexpress protrudin in a mouse model and demonstrated that such overexpression provided a partial rescue of the seizure phenotype in both pentylenetetrazole (PTZ) and intra-hippocampal kainic acid injection (IHKA) SE mouse models.

Moreover, protrudin overexpression produced a significant decrease in spontaneous action potentials in pyramidal neurons, and a significant increase in the amplitude of mIPSCs and spontaneous inhibitory postsynaptic currents (sIPSCs). Using drugs that selectively block tonic inhibition, these same investigators demonstrated that protrudin overexpression increased the amplitude of sIPSC’s in both tonic and fast synaptic signaling. Additionally, they found that while protrudin did not impact overall levels of β2 or β3-containing GABA_A_Rs, it resulted in greater localization of these receptors to the postsynaptic membrane. The functional response to altering protrudin levels likely stems from its role in exocytosis, potentially through its physical interactions with the GABA_A_R and GABARAP (Lu et al., 2019). As discussed above, NLGN2, is located on the post-synaptic membrane and thus serves to stabilize GABA_A_Rs specifically at synapses (Varoqueaux et al., 2004, 2006; Poulopoulos et al., 2009; Zhang et al., 2015). In contrast, there are also signaling pathways and interacting partners that promote dispersion of GABA_A_Rs from their synaptic localization. Excitatory signaling through NMDA receptors, which causes a calcium influx and activation of the phosphatase calcineurin, leads to the dispersion of GABA_A_Rs from the synapse via calcineurin’s dephosphorylation of key residues on the γ2 subunit of membrane GABA_A_Rs, promoting their lateral mobility (Muir et al., 2010).

While not as much is known about the proteins that promote extrasynaptic localization of GABA_A_Rs, some important progress has been made. In a study focusing on α5-subunit containing GABA_A_Rs that are normally located extrasynaptically, Torben and colleagues describe an anchoring protein, radixin, that typically serves to keep α5-containing GABA_A_Rs at the extrasynaptic membrane by preventing lateral diffusion. However, dephosphorylation of radixin, via Ras homologue family member A (RhoA)/Rho-associated protein kinase (ROCK)-mediated signaling disrupts the interaction between α5 and radixin, allowing α5-containing receptors to diffuse to the synapse as needed (Figure 1B; Hausrat et al., 2015). Thus, radixin and its interaction with α5 allow for restriction and release of α5 subunit containing GABA_A_Rs from their extrasynaptic location (Hausrat et al., 2015). Another recently described regulator of extrasynaptic GABA_A_Rs, specifically α5-subunit containing GABA_A_Rs, is the GABA_A_R auxiliary subunit Shisa Family Member 7 (Shisa7). The role of Shisa7 at inhibitory synapses was discovered incidentally when authors were trying to study the role of Shisa6–9 at excitatory synapses (Han et al., 2019). Since this initial discovery, Shisa7 has been found to play a role in both tonic and synaptic inhibition. In tonic inhibition, Shisa7 plays a critical role in the strength of tonic inhibition currents, an effect that is mediated by Shisa7’s influence on localization of α5-subunit containing GABA_A_Rs to the cell surface (Wu et al., 2021b). Specifically, Shisa7 is needed for exocytosis of GABA_A_Rs. Shisa7 is also critical for the dynamic regulation of the strength of inhibitory signaling and plays a role in homeostatic plasticity at inhibitory synapses, which is significantly impaired in mice with neuronal Shisa7 KO (Wu et al., 2021b).

## Modulation of GABAR Subunit Composition and Associated Channel Properties

While the most dynamic regulation of inhibitory signaling in the brain may occur via a change in the trafficking, stabilization, and/or localization of GABA_A_Rs, synaptically or extrasynaptically, a variation in the kind and/or levels of certain GABA_A_R subunits may have a powerful impact on long-term changes in GABA_A_R function that are associated with disease. The relative levels of various GABA_A_R subunits impacts the formation of new GABA_A_Rs (or their failure to ever assemble and leave the ER), and the ultimate properties and localization of their resulting ligand-gated channels. However, even after the synthesis of new GABA_A_R subunits, there are several additional layers of regulation at play that influence their ultimate functional contribution, and thus whether alterations in the transcript levels of unique GABA_A_R subunits will ultimately impact inhibitory transmission of the nervous system is still an active area of investigation.

As briefly discussed above, the subunit makeup of GABA_A_Rs can play a critical role in the localization, and thus the resulting function of those receptors. Synaptic receptors are capable of mediating fast phasic synaptic transmission, whereas generation of additional receptors that localize to extrasynaptic spaces, especially α5γ and α4δ-containing GABA_A_Rs, will alter tonic inhibition. On a more refined level, changes to the subunit makeup of GABA_A_Rs can influence what endogenous and exogenous modulators exert effects on the receptors, and even impact the dynamics of the resulting IPSC’s produced with opening of the chloride channels. For detailed review of the major effects of subunit composition on GABA_A_R function please see the earlier reviews of Rabow et al. and Fisher and Macdonald (Rabow et al., 1995; Fisher and Macdonald, 1997).

Alteration in the expression of GABA_A_R subunit genes (*GABRs*) were some of the earliest transcriptomic datasets to be examined in epilepsy, both in resected patient tissue and animal models. Since the identification of these changes nearly 3 decades ago, significant work has illuminated the intracellular pathways that associate with epileptogenesis. To date, a range of molecular pathways have been implicated in epilepsy, from the Janus kinase (JAK)/Signal transducer and activator of transcription (STAT) pathways to tropomyosin receptor kinase B (TrkB) and its neurotrophin brain derived neurotrophic factor (BDNF), REV-Erb alpha (Rev-Erbα), mammalian target of rapamycin (mTOR), among others (Kim et al., 1997, 2021; Tanaka et al., 1997; Brandon et al., 2000; Terunuma et al., 2008; Vaz et al., 2008; Weston et al., 2014; Zhang et al., 2021). Importantly, there is reason to believe that disruptions in many of these intracellular pathways are functionally relevant to the development or maintenance of epilepsy, as mutations in some of these pathways have been associated with epileptic syndromes (Kang, 2017).

Various studies conducted over the past two decades have provided us with a clearer understanding of how differential expression of heterogenous receptor subunits can define dynamic properties of GABA_A_Rs. In a 2014 study by Dixon et al. (2014), the authors used engineered synapses with specific subunit composition, as well as pharmacological compounds, to examine receptors containing α1, α2, γ1, and γ2 subunits in different combinations. Using macropatch currents and synaptic current measurements, they found that the identity of the α subunit of the receptors influenced the rate at which receptors deactivated after stimulation, with α2 subunit-containing receptors displaying slower deactivation. Importantly, they demonstrated that this slower deactivation came about because α2 subunit-containing receptors had a greater affinity for GABA. This may have important physiologic implications, as with less GABA release, α2 subunit-containing receptors will be easier to activate and generate an inhibitory signal then α1 subunit-containing receptors (Dixon et al., 2014). Additionally, this study demonstrated γ1-containing receptors had a slower rise in the amplitude of inhibitory current, suggesting that the receptors were not as concentrated at synapses as γ2-containing receptors. The γ subunit composition of GABA_A_Rs also has important implications for the modulation of GABA_A_Rs by benzodiazepines, as γ1 subunit-containing receptors are less responsive to benzodiazepines than γ2-containing receptors (Hanson and Czajkowski, 2008; Keramidas and Harrison, 2010).

The dynamics of fast synaptic inhibitory transmission are also influenced by the relative clustering or diffusivity of GABA_A_Rs at the postsynaptic membrane/in the synapse as well as the subunit composition of the receptors that lead to changes in kinetic properties of GABA_A_Rs and the IPSC’s thus produced (Okada et al., 2000; Schofield and Huguenard, 2007; Eyre et al., 2012). Any alterations in subunit composition which may influence the clustering of GABA_A_Rs, therefore, can change the properties of the associated receptors and synapses. Interestingly, research has demonstrated that compared to other α subunits, the α2 subunit has lower affinity for gephyrin, which would impact its clustering properties (Maric et al., 2011).

Alterations in subunit composition of GABA_A_Rs is of direct relevance to epilepsy, as reflected by two lines of evidence. First, mutations in various subunit genes, including α1, β1-β3, γ2, and δ, are the primary ligand-gated ion channel genes found to be disrupted in cases of genetic epilepsy (Kang, 2017). More recent studies have found mutations in α5 specifically in cases of epileptic encephalopathy (Hernandez et al., 2019). Additionally, several decades of studies in animal models, and more recently in tissue resected from human cases of TLE, report alterations in the levels of varying receptor subunits, which may represent compensatory responses or alternatively, pathological changes that serve to contribute to epileptogenesis (González et al., 2013, 2015; Sperk et al., 2021). Mixed results, however, are reported in the literature that may reflect differences in the particular epilepsy model that was used and/or the time of data collection since the onset of SE.

Taken together, studies employing the pilocarpine (PILO) model of SE-induced TLE in mice, and rats, differ in which GABA_A_R subunit, and subunit mRNAs, have alterations in expression. This lack of reproducibility may depend on the time elapsed since SE, and the region of brain examined. Overall, however, most studies examining levels of α5 and delta subunits, typically associated with tonic inhibition, report these subunits to be downregulated across most regions of the hippocampus and even in the entorhinal cortex (EC) and subiculum in the long-term after SE, once spontaneous seizures arise in these models (Tsunashima et al., 1997; Houser and Esclapez, 2003; Peng et al., 2004; Nishimura et al., 2005; Zhang et al., 2007; Drexel et al., 2013). Additionally, numerous studies examining expression levels between 1-90 days post-SE have found increased levels of α4 and γ2 subunits, and a decrease in α1 subunit expression, as well as an altered localization of γ2-containing receptors to perisynaptic locations (Peng et al., 2004; Zhang et al., 2007; González et al., 2015). In some studies, the increased levels of γ2 and α4 transcripts and protein seem to be maintained in the chronic stages, but in others they do not (Tsunashima et al., 1997; Drexel et al., 2013; González et al., 2013). Alterations in β1-β3 transcript and protein levels, as well as additional α subunits, have been described across numerous studies, but display less consistent patterns of change (Schwarzer et al., 1997; Tsunashima et al., 1997; Nishimura et al., 2005; Drexel et al., 2013; González et al., 2013, 2015). Results from studies conducted in tissue resected from human TLE patients undergoing surgical resection as a treatment for epilepsy, have also been varied and sometimes inconsistent with findings from animal models (Shumate et al., 1998; Pirker et al., 2003; Stefanits et al., 2019; Sperk et al., 2021). However, it may be important to keep in mind that in studies conducted using animal models, even with tissue harvested from animals with “chronic” epilepsy, the duration and severity of epilepsy, and the resultant remodeling of neural circuits, is likely less extensive and variable than the alterations present in resected human tissue. These cases represent only the most severe forms of human TLE that are insufficiently responsive to anti-seizure drug therapies.

Given the significant implication of subunit composition to the functional properties of GABA_A_Rs, along with clear evidence demonstrating alterations in mRNA levels and protein expression of numerous receptor subunits in animal and human TLE cases, an important area of past and current research exists for the development of new epilepsy therapeutics based on identifying the intracellular pathways that contribute to the genome expression of *GABRs*. Work from our own lab, in collaboration with our colleagues in the Brooks-Kayal lab, has helped to identify the intracellular signaling that leads to the transcriptional alterations in α1 and α4 gene expression in the context of epilepsy (Brünig et al., 2001; Hu et al., 2008; Lund et al., 2008). We were the first to show that these genes are differentially regulated by BDNF and that their levels are altered in the PILO model of TLE in response to increased BDNF release that is the immediate aftermath of SE (Roberts et al., 2006; Lund et al., 2008). Functionally, this leads to an increased presence of α4γ2 containing receptors in hippocampal neurons that replaces α1γ2 (Lund et al., 2008).

A unique property of GABA_A_Rs is the negative feedback regulation of their levels that occurs upon chronic exposure to GABA, which also produces an uncoupling of the receptor response to positive modulation by benzodiazepines, first described by Farb and colleagues in 1990 (Roca et al., 1990). While downregulation of GABA_A_R levels in response to GABA can be the product of complex intracellular pathways for endocytosis and degradation, as discussed above, it can also occur directly at the level of gene promoter engagement as was shown for *GABRB1* through a binding activity at the initiator sequence InR that contains the transcriptional start site (Russek et al., 2000). Recognition of this site by the polycomblike protein PHF1b and its association with Suz12 suggests that *GABRs* may be epigenetically regulated via InRs that act as sensors for overactive GABA signaling (Saha et al., 2013). Such chromatin regulation in additional subunit genes and its relationship to disorders such as epilepsy remain to be described.

**FIGURE 2:**
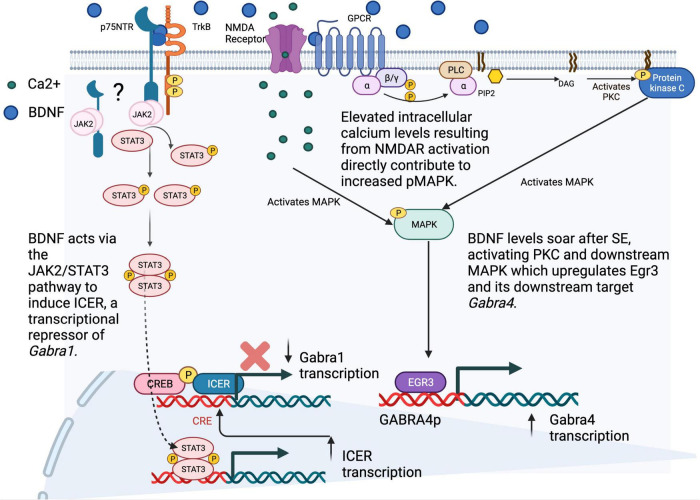
SE-induced alterations in the transcription of GABAR subunit genes. Following status epilepticus (SE), our group and others have described an increase in the transcription of the α4 subunit gene (*GABRA4*), and a decrease in the transcription of *GABRA1*. Investigations into the mechanisms behind these transcriptomic alterations have shown (on right) increased levels of BDNF after SE that lead to PKC/MAPK activation and increased levels of the immediate early gene transcription factor Egr3, both transcript and protein, as well as increased binding of Egr3 to the *GABRA4* core promoter region and (on bottom left) BDNF-induced activation of the JAK2/STAT3 pathway, through TrkB receptors (dependent also on p75NTR (remains unknown whether p75NTR is at membrane or intracellular), leading to STAT3 phosphorylation and binding of pSTAT3 homodimers to the *ICER* promoter, resulting in increased *ICER* transcription. Binding of the ICER repressor with CREB/pCREB to the *GABRA1* promoter results in decreased transcription of *GABRA1* and α1 subunit levels. Ultimately, these changes lead to an increase in the presence of α4 - containing GABA_A_Rs and a decrease in α1-containing GABA_A_Rs at the synapse, which may contribute to epileptogenesis in the post-SE setting. Figure generated using BioRender.com.

In contrast, the core promoter of *GABRA1 (GABRAp)* through sequences upstream of the InR can be upregulated via the binding of the c-AMP response element binding protein (CREB) in response to protein kinase C signaling that solidifies its role in learning and memory, as well as in synaptic plasticity (Hu et al., 2008). However, even when CREB is bound to *GABRA1p*, expression of the inducible c-AMP early repressor (ICER) can convert the “On” response to “Off”. Interestingly, this can occur in response to c-AMP signaling or in the case of BDNF, through a specific activation of the Janus Kinase (JAK)/signal transducer and activator of transcription (STAT) pathway that is dependent on TrkB (Figure 2; Raol et al., 2006; Lund et al., 2008). In parallel, BDNF signaling upregulates *GABRA4p* and GABAR subunit α4 transcript and protein levels through induction of early growth response factor 3 (EGR3) via MAPK signaling (Roberts et al., 2005, 2006). Increases in both α4 and Egr3 have been reported during spontaneous seizures weeks after SE and accompany increases in BDNF (Grabenstatter et al., 2014). While much is known about the JAK/STAT pathway in glia, its novel role in neurons remains to be determined (Nicolas et al., 2013). Current work in our labs is focused on this mystery using mouse genetics to unravel the relationship of JAK/STATs to other signaling pathways that control inhibitory neurotransmission via expression of the mammalian genome.

## Inhibitory Synapse Formation and Elimination

Another key regulator in the control of inhibitory signaling in the CNS is modulation of the formation, number/efficacy, and elimination of inhibitory synapses. The efficacy and number of inhibitory synapses plays an important role in how effective inhibitory signaling in the brain is at restraining runaway excitation. In fact, many of the genetic epilepsies that have been identified involve mutations that impact the function of proteins or channels that play an important role at inhibitory synapses. SCN1A, a sodium channel present in both excitatory and inhibitory neurons, is of particular relevance to epilepsy given that 80% of patients with Dravet syndrome, which presents with severe epilepsy in children, have loss of function *SCN1A* mutations (Marini et al., 2011). In mutant mice engineered with the human *SCN1A* mutation, heterozygous mice develop fewer inhibitory synapses than WT, and display diminished inhibitory signaling, as evidenced by smaller IPSC’s at the few inhibitory synapses that do form (Marini et al., 2011; Uchino et al., 2021). Some genetic epilepsies alternatively result from mutations in genes whose proteins impact the efficacy of inhibitory synapses through presynaptic mechanisms (Figure 1C). Most of these proteins, including syntaxin binding protein 1 (STXBP1), proline rich transmembrane protein 2 (PRRT2), and dynamin 1 (DNM1), are part of the machinery responsible for exocytosis of neurotransmitters, including GABA. By altering the size of endocytic vesicles, or the release probability of these vesicles, mutations in these proteins can exert profound effects on the size and frequency of ISPC’s.

Formation of inhibitory synapses, including clustering of GABA_A_Rs in a restricted space, is influenced by numerous factors, including essential interactions between GABA_A_R subunit protein domains and scaffolding machinery within the cells. For instance, recent work by Nathanson et al. demonstrated the essential role of a particular sequence of amino acids (a motif) within the intracellular domain of the α2 subunit of GABA_A_Rs that promotes interactions between the receptors and their binding partners, collybistin and gephyrin, that helps cluster GABA_A_Rs to produce effective inhibitory synapses (Nathanson et al., 2019). Their evidence came from mutations to this key AA motif that prevent α2 from binding with collybistin, and ultimately impair GABA_A_R localization to inhibitory synapses, especially axo-axonic. Previously these investigators demonstrated the relevance of α2 subunit interactions with collybistin by showing that disruption of α2/collybistin interactions made mice susceptible to seizures and early death (Hines et al., 2018). In recent years, the importance of collybistin, encoded by Cdc42 Guanine Nucleotide Exchange Factor 9 (ARHGEF9), to functional inhibitory synapses has become clear with the discovery of multiple patients with ARHGEF9 mutations that present with disease syndromes including epilepsy (Shimojima et al., 2011; George et al., 2021; Hines et al., 2022). Additionally, a recent study in mice demonstrated that increased expression of collybistin results in elevated GABAergic signaling and mitigates the development of seizures in an SE model (George et al., 2021).

NLGN2, in interaction with various other molecules, is also a key player in the formation of inhibitory synapses. Early research demonstrated impaired inhibitory synapse formation in mice lacking the cell adhesion molecule, NLGN2, yet only recently did researchers begin further defining which interacting partners of NLGN2 contribute to its role in synaptogenesis (Lee et al., 2013; Li et al., 2017; Wu et al., 2018).

In a study using mass spectrometry to identify binding partners of NLGN2 that may be necessary to promote development of inhibitory synapses, Wu, Tian et al showed the functional relevance of LHFPL4 to inhibitory synapse formation *in vitro* by demonstrating fewer inhibitory synapses formed in cultured neurons with LHFPL4 KO (Wu et al., 2018). Additionally, they demonstrated the functional consequences of LHFPL4 KO in cerebellar neurons as mice with this deletion display impaired motor behavior and were more susceptible to seizures than WT. In a separate study examining the interaction of Neuroligin 2 and Slitrk3 in the formation of inhibitory synapses, both Neuroligin 2 and Slitrk3 (ST3) participated in the development of functional GABAergic synapses, such that mice lacking either of these cell adhesion molecules had increased seizure susceptibility and altered network activity (Li et al., 2017). Through use of a combination of genetic KO models and RNAi- mediated knockdown (KD), Li et al. demonstrated an essential role for NLGN2, specifically in the *development* of inhibitory synapses, but after neurons mature, there is some recovery in inhibitory neurotransmission even in NLGN2 KO mice. shRNA-KD of NLGN2 at 2 days *in vitro* (DIV2) significantly decreased GABAergic neurotransmission at DIV8, whereas shRNA-KD of ST3 at DIV2 did not, suggesting that NLGN2 is essential at that time but ST3 is not. Later in development, elimination of either ST3 or NLGN2 at DIV10 significantly decreased mIPSCs at DIV18, representative of more mature neurons, and the effects of KO were additive. MAM Domain Containing Glycosylphosphatidylinositol Anchor proteins (MDGAs), on the other hand, inhibit the role of NLGN2 in forming inhibitory synapses, as KD of these molecules in the adult brain led to increased inhibitory synapse formation and upregulation decreased the number of inhibitory synapses (Lee et al., 2013). MDGAs that interact with NGLN2 interact with NGLN2 in a cis formation (are on the same side of the synapse, postsynaptic).

Generation of inhibitory synapses is also regulated at the level of transcription, with neuronal PAS domain protein 4 (NPAS4) serving as a key transcription factor that is responsive to local neuron activity levels and plays a role in determining the number of functional inhibitory synapses that are formed (Lin et al., 2008). Another recent study by Gu and colleagues illustrated the relevance of neuronal activity in promoting the development of inhibitory synapses, and provided one potential mechanism for the maintenance of excitatory/inhibitory balance in development. Using a mouse model with sparsely expressed, Cre-dependent KO of GLUN1 and GLUA1-3 in select neurons (totaling less than 1% of total neurons), they were able to track Cre-expressing neurons and study, at a single neuron level, the impact of AMPA and GLUN1 KO on the ability of select neurons to form inhibitory synapses (Gu et al., 2016). When neurons harvested from E18 embryos, and grown *in vitro* for 3–7 days, were recorded from, those lacking GLUN1 displayed decreased amplitude and frequency in synaptic mIPSCs. Additionally, acute hippocampal slices from these conditional KO mice demonstrated impaired GABAergic transmission, and immunostaining revealed reduced VGAT, gephyrin, and NLGN2 in Cre^+^ neurons.

When it comes to the elimination of inhibitory synapses, microglia are a key cell type involved in the process. During development, microglia play an essential role in synaptic pruning, disruptions of which have been implicated in various neuropsychiatric disorders, including autism and schizophrenia. In addition to this developmental function, microglia continue to play a role in refinement of synaptic connections into adulthood (Andoh et al., 2019; Park et al., 2021). In order for microglia to know which synapses to phagocytose, they look for the presence of particular “tags” on them, which in the case of inhibitory synapses, seems to be stable presentation of phosphotidylserine (PTD) on the outer surface of the plasma membrane. Normally, the phospholipid-flipase chaperone protein Cdc50a regulates Ptd exposure, and the deletion of Cdc50a was found to lead to stable Ptd expression and subsequent elimination of inhibitory synapses by MER proto-oncogene tyrosine kinase (MERTK) microglial signaling (Park et al., 2021). Importantly, the role of microglia in synaptic pruning may be particularly relevant to epilepsy, as a recent review by Andoh et al. suggests that increased and dysregulated synaptic pruning in the epileptic brain, leading to the inappropriate elimination of inhibitory synapses by microglial phagocytosis, could contribute to an emergence of E/I imbalance (Andoh et al., 2019).

## Vulnerable GABAergic Neuronal Populations

Early research into the molecular and cellular underpinnings of epilepsy focused on the potential selective loss of inhibitory neurons after insults in models of TLE. Indeed, excessive loss of inhibitory neurons compared to excitatory neurons could provide a reasonable explanation for the E/I imbalance that defines epilepsy. Early studies confirmed inhibitory neuron death in many epilepsy models, including SE models with kindling and pilocarpine (Dinocourt et al., 2003; Kobayashi and Buckmaster, 2003; Sayin et al., 2003; Dudek, 2020). However, broad inhibitory neuronal loss may cause varying phenotypes depending on the subclass of inhibitory neurons that is impacted. Early studies demonstrated that PV^+^ interneurons in the hippocampus are particularly vulnerable to insults (Bouilleret et al., 2000; Kuruba et al., 2011). Given that these PV^+^ neurons normally serve as the breaks for the key tri-synaptic circuit that projects information within and subsequently out of the hippocampus, their loss could have a profound effect on the ability of the hippocampus to dampen hyperactivity at a time when it could be managed.

One factor that is hypothesized to contribute to the differential vulnerability of specific classes of interneurons is the population of calcium binding proteins that they contain. Calcium binding proteins, including PV, calbindin (CB), and calretinin (CR), serve an important role as a buffering system for transient dysregulation in intracellular calcium levels. Thus, in the context of excessive excitatory signaling, calcium binding proteins that can buffer more calcium may provide added resilience against such insults when compared to the neuronal subtypes that primarily contain calcium binding proteins with lower buffering capacity. Of the 3 primary calcium binding proteins that are known to define distinct sets of inhibitory neurons in the hippocampus, PV has the lowest buffering capacity, while CR has the highest, which may contribute to the almost invariant vulnerability of PV^+^ interneurons observed in animal models of TLE (Dinocourt et al., 2003; Kobayashi and Buckmaster, 2003; Marx et al., 2013). And yet, even CR-expressing interneurons in the hippocampus, which should be afforded the greatest protection against calcium influxes, appear vulnerable to degeneration in both animals and humans with TLE (Tóth and Maglóczky, 2014). While CB^+^ inhibitory neurons are largely protected against degeneration in epilepsy models, whether it is because of the calcium buffering capacity of CB, or perhaps other features of the neurons expressing CB, remains to be determined (Fairless et al., 2019). In addition to these studies, research has focused on other interneuron subsets that include those defined by the expression of specific markers, including SST, neuropeptide Y (NPY), or cholecystokinin (CCK). In an IHKA mouse model of TLE, PV^+^ interneurons displayed selective vulnerability compared to NPY^+^ neurons at 2 days and 3 weeks post-SE. This effect was seen most clearly in regions of the hippocampus closest to the injection insult, including the septal and dorsal hippocampus, but not in the temporal or ventral intermediate hippocampus in these early time points (Marx et al., 2013). In contrast, a separate study conducted using the same IHKA mouse model with KA injection into the dorsal hippocampus focused specifically on CCK^+^ interneurons and found that CCK^+^ basket cells were susceptible along the entire dorsal to ventral access, not just in the regions immediately neighboring the insult (Kang et al., 2021). Taken together, the cellular responses of certain classes of neurons to a change in circuit behavior leaves them susceptible to irreparable damage and we need a better means to identify their nodes of engagement where balance can be restored.

With the advent and large-scale application of single cell technologies, future research will be poised to identify selectively vulnerable classes of inhibitory neurons, whose protection may offer a new therapeutic avenue in epilepsy (Bruzelius et al., 2021; Waloschková et al., 2021). Additionally, identification of classes of inhibitory neurons that are resilient to epileptic insults could provide insights into methods of promoting resilience in typically vulnerable inhibitory neurons, a technique which is currently being explored in the Alzheimer’s field (Pfisterer et al., 2020; Roussarie et al., 2020; Leng et al., 2021). In the first single nuclei RNA sequencing (snRNAseq) study of surgically resected tissue from the temporal lobe of patients with TLE, researchers distinguished 23 different subclusters of GABAergic neurons, and even more refined characterizations will be possible through the acquisition of larger datasets (Pfisterer et al., 2020). To begin to identify especially susceptible neurons, the authors compared the relative abundance of each of the many GABAergic subclasses that exist between human TLE and control autopsy cases, as well as determined which subtypes display the greatest shift in their gene expression profile. In agreement with much of the existing research on broad classes of GABAergic neurons, the investigators found the greatest reduction in parvalbumin, sulfatase1 (SULF1)-expressing GABAergic neurons in tissue from cases compared to controls, pointing to a specific subset of PV^+^ neurons that may be particularly susceptible to degeneration in TLE. As for transcriptomic alterations, subsets of many broad classes of inhibitory neurons, defined by the authors using known subtype-specific expression markers derived from literature, including PV^+^ neurons expressing SULF1, PV^+^ neurons expressing nitric oxide synthase 1 (NOS1), vasoactive intestinal peptide positive neurons expressing cerebellin 1 precursor, SST^+^ neurons expressing tachykinin precursor 1, and inhibitor of DNA binding 2 positive neurons expressing lysosomal associated membrane protein family member 5 and NOS1, displayed dramatic shifts between control and TLE cases, providing more detailed information than was previously possible regarding GABAergic neurons and their signaling in the setting of human chronic TLE.

In parallel with technological advances in single cell RNA-sequencing, recent advances in spatial transcriptomics are allowing researchers to capture spatially resolved single cell or even subcellular level transcriptomic and proteomic data. These spatially resolved techniques are a particularly valuable resource in epilepsy, as they will allow for an in-depth investigation of whole circuits that are disrupted and detail how alterations in one brain region may relate to those in another on an epigenetic, transcriptomic, and proteomic level. Spatial approaches that give context to transcriptomic findings may indeed provide important insights into the progression of epilepsy from the hyperactivity of neuronal subsets to a circuit level disorder in which epigenetic or genomic changes may drive epileptogenesis, providing new strategies for therapeutic intervention.

## Summary/Future Directions

Significant progress has been made in the past three decades to elucidate the various cellular mechanisms that allow for fine grained control over inhibitory signaling within the CNS. Great strides have been made in our understanding of factors regulating the formation, properties, stability, and localization of GABA_A_Rs, the primary receptors responsible for synaptic and extrasynaptic neurotransmission, as well as the formation, maintenance, and elimination of inhibitory synapses in both health and disease. In part, this progress has been informed by the genomic revolution, which in the past two decades has allowed for identification of specific gene mutations in an increased number of individuals with genetic epilepsies, the exploration of which have propelled our understanding of GABAergic signaling forward. In the same vein, the single cell and spatial transcriptomic revolutions of the past five years have already begun to illuminate advancing complexity in the identity and function of cell types throughout diverse organs, including the brain, and offer a rich opportunity for enhancing our understanding of GABAergic neurons in the context of neural circuits that are dysregulated in a plethora of brain disorders, including epilepsy. The promise of multiomics to bring together the regulation of the genome with the functional proteomic landscape that is carved into the brain’s connectome will open new opportunities to rethink the development of curative therapies to restore the capacity of the brain for rebalance using its dynamic and complex inhibitory signaling properties.

## Author Contributions

AT generated the original text for the document which was edited in collaboration with SR. AT generated the figures for general information to synthesize key concepts. Both authors contributed to the article and approved the submitted version.

## Conflict of Interest

The authors declare that the research was conducted in the absence of any commercial or financial relationships that could be construed as a potential conflict of interest.

## Publisher’s Note

All claims expressed in this article are solely those of the authors and do not necessarily represent those of their affiliated organizations, or those of the publisher, the editors and the reviewers. Any product that may be evaluated in this article, or claim that may be made by its manufacturer, is not guaranteed or endorsed by the publisher.

## References

[B1] Andoh M. Ikegaya Y. Koyama R. (2019). Synaptic pruning by microglia in epilepsy. *J. Clin. Med.* 8:2170. 10.3390/jcm8122170 31818018 PMC6947403

[B2] Asadi-Pooya A. A. Stewart G. R. Abrams D. J. Sharan A. (2017). Prevalence and incidence of drug-resistant mesial temporal lobe epilepsy in the united states. *World Neurosurg*. 99 662–666. 10.1016/j.wneu.2016.12.074 28034810

[B3] Bannai H. Lévi S. Schweizer C. Inoue T. Launey T. Racine V. (2009). Activity-dependent tuning of inhibitory neurotransmission based on GABAAR diffusion dynamics. *Neuron* 62 670–682. 10.1016/j.neuron.2009.04.023 19524526

[B4] Beghi E. (2020). The epidemiology of epilepsy. *Neuroepidemiology* 54 185–191. 10.1159/000503831 31852003

[B5] Ben-Ari Y. Tremblay E. Ottersen O. P. (1980). Injections of kainic acid into the amygdaloid complex of the rat: An electrographic, clinical and histological study in relation to the pathology of epilepsy. *Neuroscience* 5 515–528. 10.1016/0306-4522(80)90049-4 6892841

[B6] Bogdanov Y. Michels G. Armstrong-Gold C. Haydon P. G. Lindstrom J. Pangalos M. (2006). Synaptic GABAA receptors are directly recruited from their extrasynaptic counterparts. *EMBO J*. 25 4381–4389. 10.1038/sj.emboj.7601309 16946701 PMC1570424

[B7] Bouilleret V. Loup F. Kiener T. Marescaux C. Fritschy J. (2000). Early loss of interneurons and delayed subunit-specific changes in GABAA-receptor expression in a mouse model of mesial temporal lobe epilepsy. *Hippocampus* 10 305–324. 10.1002/1098-1063(2000)10:3<305::AID-HIPO11>3.0.CO;2-I 10902900

[B8] Brady M. L. Pilli J. Lorenz-Guertin J. M. Das S. Moon C. E. Graff N. (2018). Depolarizing, inhibitory GABA type A receptor activity regulates GABAergic synapse plasticity via ERK and BDNF signaling. *Neuropharmacology* 128 324–339. 10.1016/j.neuropharm.2017.10.022 29074304 PMC5739058

[B9] Brandon N. J. Delmas P. Kittler J. T. McDonald B. J. Sieghart W. Brown D. A. (2000). GABAA receptor phosphorylation and functional modulation in cortical neurons by a protein kinase C-dependent pathway. *J. Biol. Chem*. 275 38856–38862. 10.1074/jbc.M004910200 10978327

[B10] Brünig I. Penschuck S. Berninger B. Benson J. Fritschy J. M. (2001). BDNF reduces miniature inhibitory postsynaptic currents by rapid downregulation of GABA(A) receptor surface expression. *Eur. J. Neurosci*. 13 1320–1328. 10.1046/j.0953-816x.2001.01506.x 11298792

[B11] Bruzelius A. Kidnapillai S. Drouin-Ouellet J. Stoker T. Barker R. A. Rylander Ottosson D. (2021). Reprogramming human adult fibroblasts into GABAergic interneurons. *Cells* 10:3450. 10.3390/cells10123450 34943958 PMC8699824

[B12] Caraiscos V. B. Elliott E. M. You-Ten K. E. Cheng V. Y. Belelli D. Newell J. G. (2004). Tonic inhibition in mouse hippocampal CA1 pyramidal neurons is mediated by alpha5 subunit-containing gamma-aminobutyric acid type A receptors. *Proc. Natl. Acad. Sci. U. S. A*. 101 3662–3667. 10.1073/pnas.0307231101 14993607 PMC373519

[B13] Chandra D. Jia F. Liang J. Peng Z. Suryanarayanan A. Werner D. F. (2006). GABAA receptor alpha 4 subunits mediate extrasynaptic inhibition in thalamus and dentate gyrus and the action of gaboxadol. *Proc. Natl. Acad. Sci. U. S. A*. 103 15230–15235. 10.1073/pnas.0604304103 17005728 PMC1578762

[B14] Chiu C. Q. Martenson J. S. Yamazaki M. Natsume R. Sakimura K. Tomita S. (2018). Input-specific NMDAR-dependent potentiation of dendritic GABAergic inhibition. *Neuron* 97 368–377.e3. 10.1016/j.neuron.2017.12.032 29346754 PMC5777295

[B15] Davenport E. C. Pendolino V. Kontou G. McGee T. P. Sheehan D. F. Lopez-Domenech G. (2017). An essential role for the tetraspanin LHFPL4 in the cell-type-specific targeting and clustering of synaptic GABAA receptors. *Cell Rep*. 21 70–83. 10.1016/j.celrep.2017.09.025 28978485 PMC5640807

[B16] Dibbens L. M. Tarpey P. S. Hynes K. Bayly M. A. Scheffer I. E. Smith R. (2008). X-linked protocadherin 19 mutations cause female-limited epilepsy and cognitive impairment. *Nat. Genet.* 40 776–781. 10.1038/ng.149 18469813 PMC2756413

[B17] Dinocourt C. Petanjek Z. Freund T. F. Ben-Ari Y. Esclapez M. (2003). Loss of interneurons innervating pyramidal cell dendrites and axon initial segments in the CA1 region of the hippocampus following pilocarpine-induced seizures. *J. Comp. Neurol*. 459 407–425. 10.1002/cne.10622 12687707

[B18] Dixon C. Sah P. Lynch J. W. Keramidas A. (2014). GABAA receptor α and γ subunits shape synaptic currents via different mechanisms. *J. Biol. Chem*. 289 5399–5411. 10.1074/jbc.M113.514695 24425869 PMC3937617

[B19] Drexel M. Kirchmair E. Sperk G. (2013). Changes in the expression of GABAA receptor subunit mRNAs in parahippocampal areas after kainic acid induced seizures. *Front. Neural Circ*. 7:142. 10.3389/fncir.2013.00142 24065890 PMC3776158

[B20] Dudek F. E. (2020). Loss of GABAergic interneurons in seizure-induced Epileptogenesis—Two decades later and in a more complex world. *Epilepsy Curr.* 20 70S–72S. 10.1177/1535759720960464 33059465 PMC7726724

[B21] Eckel R. Szulc B. Walker M. C. Kittler J. T. (2015). Activation of calcineurin underlies altered trafficking of α2 subunit containing GABAA receptors during prolonged epileptiform activity. *Neuropharmacology* 88 82–90. 10.1016/j.neuropharm.2014.09.014 25245802 PMC4239296

[B22] Epilepsy data and statistics CDC (2020). *Epilepsy data and statistics | CDC.* Available online at: https://www.cdc.gov/epilepsy/data/index.html [accessed on Feb 14, 2022].

[B23] Epilepsy (2022). *Epilepsy.* Available online at: https://www.who.int/news-room/fact-sheets/detail/epilepsy [accessed on Aprl 04, 2022]

[B24] Essrich C. Lorez M. Benson J. A. Fritschy J. M. Lüscher B. (1998). Postsynaptic clustering of major GABAA receptor subtypes requires the gamma 2 subunit and gephyrin. *Nat. Neurosci.* 1 563–571. 10.1038/2798 10196563

[B25] Eyre M. D. Renzi M. Farrant M. Nusser Z. (2012). Setting the time course of inhibitory synaptic currents by mixing multiple GABA(A) receptor α subunit isoforms. *J. Neurosci*. 32 5853–5867. 10.1523/JNEUROSCI.6495-11.2012 22539847 PMC3348502

[B26] Fairless R. Williams S. K. Diem R. (2019). Calcium-binding proteins as determinants of central nervous system neuronal vulnerability to disease. *Int. J. Mol. Sci.* 20:2146. 10.3390/ijms20092146 31052285 PMC6539299

[B27] Fisher J. L. Macdonald R. L. (1997). Single channel properties of recombinant GABAA receptors containing gamma 2 or delta subtypes expressed with alpha 1 and beta 3 subtypes in mouse L929 cells. *J. Physiol*. 505 283–297. 10.1111/j.1469-7793.1997.283bb.x 9423172 PMC1160063

[B28] Flores C. E. Nikonenko I. Mendez P. Fritschy J. Tyagarajan S. K. Muller D. (2015). Activity-dependent inhibitory synapse remodeling through gephyrin phosphorylation. *Proc. Natl. Acad. Sci. U. S. A.* 112 E65–E72. 10.1073/pnas.1411170112 25535349 PMC4291629

[B29] Fricke S. Metzdorf K. Ohm M. Haak S. Heine M. Korte M. (2019). Fast regulation of GABAAR diffusion dynamics by nogo-A signaling. *Cell Rep.* 29 671–684.e6. 10.1016/j.celrep.2019.09.015 31618635

[B30] Fritschy J. (2008). Epilepsy, E/I balance and GABA(A) receptor plasticity. *Front. Mole. Neurosci*. 1:5. 10.3389/neuro.02.005.2008 18946538 PMC2525999

[B31] Garcia J. D. Gookin S. E. Crosby K. C. Schwartz S. L. Tiemeier E. Kennedy M. J. (2021). Stepwise disassembly of GABAergic synapses during pathogenic excitotoxicity. *Cell Rep*. 37:110142. 10.1016/j.celrep.2021.110142 34936876 PMC8824488

[B32] Ge Y. Kang Y. Cassidy R. M. Moon K.-M. Lewis R. Wong R. O. L. (2018). Clptm1 limits forward trafficking of GABAA receptors to scale inhibitory synaptic strength. *Neuron* 97 596–610.e8. 10.1016/j.neuron.2017.12.038 29395912 PMC5810584

[B33] George S. James S. De Blas A. L. (2021). Selective overexpression of collybistin in mouse hippocampal pyramidal cells enhances GABAergic neurotransmission and protects against PTZ-induced seizures. *eNeuro* 8 ENEURO.561–ENEURO.520. 10.1523/ENEURO.0561-20.2021 34083383 PMC8281261

[B34] Ghosh H. Auguadri L. Battaglia S. Thirouin Z. S. Zemoura K. Messner S. (2016). Several posttranslational modifications act in concert to regulate gephyrin scaffolding and GABAergic transmission. *Nat. Commun.* 7:13365. 10.3929/ethz-b-000122567 PMC5103071 27819299

[B35] Glykys J. Mann E. O. Mody I. (2008). Which GABA(A) receptor subunits are necessary for tonic inhibition in the hippocampus? *J. Neurosci*. 28 1421–1426. 10.1523/JNEUROSCI.4751-07.2008 18256262 PMC6671570

[B36] Gonzalez M. I. (2013). The possible role of GABAA receptors and gephyrin in epileptogenesis. *Front. Cell Neurosci.* 7:113. 10.3389/fncel.2013.00113 23885234 PMC3717475

[B37] González M. I. (2019). Calpain-dependent cleavage of GABAergic proteins during epileptogenesis. *Epilepsy Res.* 157:106206. 10.1016/j.eplepsyres.2019.106206 31585309 PMC6897327

[B38] González M. I. Angel Y. C. D. Brooks-Kayal A. (2013). Down-regulation of gephyrin and GABAA receptor subunits during epileptogenesis in the CA1 region of hippocampus. *Epilepsia* 54 616–624. 10.1111/epi.12063 23294024 PMC3618570

[B39] González M. I. Grabenstatter H. L. Cea-Del Rio C. A. Angel Y. Carlsen J. Laoprasert R. P. (2015). Seizure-related regulation of GABAA receptors in spontaneously epileptic rats. *Neurobiol. Dis*. 77 246–256. 10.1016/j.nbd.2015.03.001 25769812 PMC4415851

[B40] Grabenstatter H. L. Cogswell M. Cruz Del, Angel Y. Carlsen J. Gonzalez M. I. (2014). Effect of spontaneous seizures on GABAA receptor α4 subunit expression in an animal model of temporal lobe epilepsy. *Epilepsia* 55 1826–1833. 10.1111/epi.12771 25223733 PMC4596257

[B41] Griffin C. E. Kaye A. M. Bueno F. R. Kaye A. D. (2013). Benzodiazepine pharmacology and central nervous System–Mediated effects. *Ochsner J*. 13 214–223. 23789008 PMC3684331

[B42] Gu X. Zhou L. Lu W. (2016). An NMDA receptor-dependent mechanism underlies inhibitory synapse development. *Cell Rep.* 14 471–478. 10.1016/j.celrep.2015.12.061 26774487 PMC4765167

[B43] Han W. Li J. Pelkey K. A. Pandey S. Chen X. Wang Y.-X. (2019). Shisa7 is a GABAA receptor auxiliary subunit controlling benzodiazepine actions. *Science* 366 246–250. 10.1126/science.aax5719 31601770 PMC7382361

[B44] Hannan S. Minere M. Harris J. Izquierdo P. Thomas P. Tench B. (2020). GABAAR isoform and subunit structural motifs determine synaptic and extrasynaptic receptor localisation. *Neuropharmacology* 169:107540. 10.1016/j.neuropharm.2019.02.022 30794836

[B45] Hanson S. M. Czajkowski C. (2008). Structural mechanisms underlying benzodiazepine modulation of the GABAA receptor. *J. Neurosci*. 28 3490–3499. 10.1523/JNEUROSCI.5727-07.2008 18367615 PMC2410040

[B46] Hausrat T. J. Muhia M. Gerrow K. Thomas P. Hirdes W. Tsukita S. (2015). Radixin regulates synaptic GABA(A) receptor density and is essential for reversal learning and short-term memory. *Nat. Commun.* 6:6872. 10.3929/ethz-b-000100979 PMC4411296 25891999

[B47] Hernandez C. C. XiangWei W. Hu N. Shen D. Shen W. Lagrange A. H. (2019). Altered inhibitory synapses in de novo GABRA5 and GABRA1 mutations associated with early onset epileptic encephalopathies. *Brain* 142 1938–1954. 10.1093/brain/awz123 31056671 PMC6598634

[B48] Hines D. J. Contreras A. Garcia B. Barker J. S. Boren A. J. El Achkar C. (2022). Human ARHGEF9 intellectual disability syndrome is phenocopied by a mutation that disrupts collybistin binding to the GABAA receptor α2 subunit. *Mol. Psychiatry* 27 1729–1741. 10.1038/s41380-022-01468-z 35169261 PMC9095487

[B49] Hines R. M. Davies P. A. Moss S. J. Maguire J. (2012). Functional regulation of GABAA receptors in nervous system pathologies. *Curr. Opin. Neurobiol*. 22 552–558. 10.1016/j.conb.2011.10.007 22036769 PMC3846183

[B50] Hines R. M. Maric H. M. Hines D. J. Modgil A. Panzanelli P. Nakamura Y. (2018). Developmental seizures and mortality result from reducing GABAA receptor α2-subunit interaction with collybistin. *Nat. Commun*. 9:3130. 10.1038/s41467-018-05481-1 30087324 PMC6081406

[B51] Houser C. R. Esclapez M. (2003). Downregulation of the α5 subunit of the GABAA receptor in the pilocarpine model of temporal lobe epilepsy. *Hippocampus* 13 633–645. 12921352 10.1002/hipo.10108

[B52] Hu Y. Lund I. V. Gravielle M. C. Farb D. H. Brooks-Kayal A. R. Russek S. J. (2008). Surface expression of GABAA receptors is transcriptionally controlled by the interplay of cAMP-response element-binding protein and its binding partner inducible cAMP early repressor. *J. Biol. Chem*. 283 9328–9340. 10.1074/jbc.M705110200 18180303 PMC2431045

[B53] Huang X. Hernandez C. C. Hu N. Macdonald R. L. (2014). Three epilepsy-associated GABRG2 missense mutations at the γ+/β- interface disrupt GABAA receptor assembly and trafficking by similar mechanisms but to different extents. *Neurobiol. Dis*. 68 167–179. 10.1016/j.nbd.2014.04.015 24798517 PMC4169075

[B54] Jacob T. C. Bogdanov Y. D. Magnus C. Saliba R. Kittler J. Haydon P. G. (2005). Gephyrin regulates the cell surface dynamics of synaptic GABAA receptors. *J. Neurosci.* 25 10469–10478. 10.1523/JNEUROSCI.2267-05.2005 16280585 PMC6725824

[B55] Joshi S. Rajasekaran K. Hawk K. M. Brar J. Ross B. M. Tran C. A. (2015). Phosphatase inhibition prevents the activity-dependent trafficking of GABAA receptors during status epilepticus in the young animal. *Epilepsia* 56 1355–1365. 10.1111/epi.13098 26248944

[B56] Kalbouneh H. Schlicksupp A. Kirsch J. Kuhse J. (2014). Cyclin-dependent kinase 5 is involved in the phosphorylation of gephyrin and clustering of GABAA receptors at inhibitory synapses of hippocampal neurons. *PLoS One* 9:e104256. 10.1371/journal.pone.0104256 25093719 PMC4122414

[B57] Kang J. (2017). Defects at the crossroads of GABAergic signaling in generalized genetic epilepsies. *Epilepsy Res*. 137 9–18. 10.1016/j.eplepsyres.2017.08.013 28865303 PMC6112605

[B58] Kang J. Shen W. Macdonald R. L. (2013). Trafficking-deficient mutant gabrg2 subunit amount may modify epilepsy phenotype. *Ann. Neurol*. 74 547–559. 10.1002/ana.23947 23720301 PMC3839255

[B59] Kang Y. Clement E. M. Park I. Greenfield L. J. Smith B. N. Lee S. (2021). Vulnerability of cholecystokinin-expressing GABAergic interneurons in the unilateral intrahippocampal kainate mouse model of temporal lobe epilepsy. *Exp. Neurol*. 342:113724. 10.1016/j.expneurol.2021.113724 33915166 PMC8192495

[B60] Keezer M. R. Sisodiya S. M. Sander J. W. (2016). Comorbidities of epilepsy: Current concepts and future perspectives. *Lancet Neurol*. 15 106–115. 10.1016/S1474-4422(15)00225-2 26549780

[B61] Keramidas A. Harrison N. L. (2010). The activation mechanism of α1β2γ2S and α3β3γ2S GABAA receptors. *J. General Physiol.* 135 59–75. 10.1085/jgp.200910317 20038526 PMC2806416

[B62] Kim D. Jun K. S. Lee S. B. Kang N.-G. Min D. K. Kim Y.-H. (1997). Phospholipase C isozymes selectively couple to specific neurotransmitter receptors. *Nature* 389 290–293. 10.1038/38508 9305844

[B63] Kim H. Y. Suh P. Kim J. (2021). The role of phospholipase C in GABAergic inhibition and its relevance to epilepsy. *Int. J. Mol. Sci.* 22:3149. 10.3390/ijms22063149 33808762 PMC8003358

[B64] Kins S. Betz H. Kirsch J. (2000). Collybistin, a newly identified brain-specific GEF, induces submembrane clustering of gephyrin. *Nat. Neurosci.* 3 22–29. 10.1038/71096 10607391

[B65] Kittler J. T. Thomas P. Tretter V. Bogdanov Y. D. Haucke V. Smart T. G. (2004). Huntingtin-associated protein 1 regulates inhibitory synaptic transmission by modulating -aminobutyric acid type A receptor membrane trafficking. *Proc. Natl. Acad. Sci. U. S. A.* 101 12736–12741. 10.1073/pnas.0401860101 15310851 PMC515122

[B66] Kneussel M. Brandstätter J. H. Gasnier B. Feng G. Sanes J. R. Betz H. (2001). Gephyrin-independent clustering of postsynaptic GABA(A) receptor subtypes. *Mol. Cell Neurosci.* 17 973–982. 10.1006/mcne.2001.0983 11414787

[B67] Kneussel M. Brandstätter J. H. Laube B. Stahl S. Müller U. Betz H. (1999). Loss of postsynaptic GABA(A) receptor clustering in gephyrin-deficient mice. *J. Neurosci*. 19 9289–9297. 10.1523/JNEUROSCI.19-21-09289.1999 10531433 PMC6782938

[B68] Kobayashi M. Buckmaster P. S. (2003). Reduced inhibition of dentate granule cells in a model of temporal lobe epilepsy. *J. Neurosci*. 23 2440–2452. 12657704 10.1523/JNEUROSCI.23-06-02440.2003 PMC6741996

[B69] Kolc K. L. Sadleir L. G. Scheffer I. E. Ivancevic A. Roberts R. Pham D. H. (2019). A systematic review and meta-analysis of 271 PCDH19-variant individuals identifies psychiatric comorbidities, and association of seizure onset and disease severity. *Mole. Psych*. 24 241–251. 10.1038/s41380-018-0066-9 29892053 PMC6344372

[B70] Kuruba R. Hattiangady B. Parihar V. K. Shuai B. Shetty A. K. (2011). Differential susceptibility of interneurons expressing neuropeptide Y or parvalbumin in the aged hippocampus to acute seizure activity. *PLoS One* 6:e24493. 10.1371/journal.pone.0024493 21915341 PMC3167860

[B71] Lagrange A. H. Botzolakis E. J. Macdonald R. L. (2007). Enhanced macroscopic desensitization shapes the response of alpha4 subtype-containing GABAA receptors to synaptic and extrasynaptic GABA. *J. Physiol*. 578 655–676. 10.1113/jphysiol.2006.122135 17124266 PMC2151343

[B72] Lee K. Kim Y. Lee S. Qiang Y. Lee D. Lee H. W. (2013). MDGAs interact selectively with neuroligin-2 but not other neuroligins to regulate inhibitory synapse development. *Proc. Natl. Acad. Sci. U. S. A.* 110 336–341. 10.1073/pnas.1219987110 23248271 PMC3538197

[B73] Leng K. Li E. Eser R. Piergies A. Sit R. Tan M. (2021). Molecular characterization of selectively vulnerable neurons in alzheimer’s disease. *Nat. Neurosci*. 24 276–287. 10.1038/s41593-020-00764-7 33432193 PMC7854528

[B74] Lévi S. Logan S. M. Tovar K. R. Craig A. M. (2004). Gephyrin is critical for glycine receptor clustering but not for the formation of functional GABAergic synapses in hippocampal neurons. *J. Neurosci.* 24 207–217. 10.1523/JNEUROSCI.1661-03.2004 14715953 PMC6729579

[B75] Li J. Han W. Pelkey K. A. Duan J. Mao X. Wang Y.-X. (2017). Molecular dissection of neuroligin 2 and Slitrk3 reveals an essential framework for GABAergic synapse development. *Neuron* 96 808–826.e8. 10.1016/j.neuron.2017.10.003 29107521 PMC5957482

[B76] Lin Y. Bloodgood B. L. Hauser J. L. Lapan A. D. Koon A. C. Kim T.-K. (2008). Activity-dependent regulation of inhibitory synapse development by Npas4. *Nature* 455 1198–1204. 10.1038/nature07319 18815592 PMC2637532

[B77] Lorenz-Guertin J. M. Bambino M. J. Jacob T. C. (2018). Γ2 GABAAR trafficking and the consequences of human genetic variation. *Front. Cell Neurosci*. 12:265. 10.3389/fncel.2018.00265 30190672 PMC6116786

[B78] Löscher W. Klein P. (2021). The pharmacology and clinical efficacy of antiseizure medications: From bromide salts to cenobamate and beyond. *CNS Drugs* 35 935–963. 10.1007/s40263-021-00827-8 34145528 PMC8408078

[B79] Lu X. Yang Y. Zhou R. Li Y. Yang Y. Wang X. (2019). Protrudin modulates seizure activity through GABAA receptor regulation. *Cell Death Dis*. 10:897. 10.1038/s41419-019-2118-8 31772151 PMC6879747

[B80] Lund I. V. Hu Y. Raol Y. H. Benham R. S. Faris R. Russek S. J. (2008). BDNF selectively regulates GABAA receptor transcription by activation of the JAK/STAT pathway. *Sci. Signal.* 1:ra1. 10.1126/scisignal.1162396 18922788 PMC2651003

[B81] Luscher B. Fuchs T. Kilpatrick C. L. (2011). GABAAR trafficking-mediated plasticity of inhibitory synapses. *Neuron* 70 385–409. 10.1016/j.neuron.2011.03.024 21555068 PMC3093971

[B82] Maric H. Mukherjee J. Tretter V. Moss S. J. Schindelin H. (2011). Gephyrin-mediated γ-aminobutyric acid type A and glycine receptor clustering relies on a common binding site. *J. Biol. Chem*. 286 42105–42114. 10.1074/jbc.M111.303412 22006921 PMC3234978

[B83] Marini C. Scheffer I. E. Nabbout R. Suls A. Jonghe P. D. Zara F. (2011). The genetics of dravet syndrome. *Epilepsia* 52 24–29. 10.1111/j.1528-1167.2011.02997.x 21463275

[B84] Marsden K. C. Beattie J. B. Friedenthal J. Carroll R. C. (2007). NMDA receptor activation potentiates inhibitory transmission through GABA receptor-associated protein-dependent exocytosis of GABA(A) receptors. *J. Neurosci*. 27 14326–14337. 10.1523/JNEUROSCI.4433-07.2007 18160640 PMC6673443

[B85] Marshall C. R. Young E. J. Pani A. M. Freckmann M.-L. Lacassie Y. Howald C. (2008). Infantile spasms is associated with deletion of the MAGI2 gene on chromosome 7q11.23-q21.11. *Am. J. Hum. Genetics* 83 106–111. 10.1016/j.ajhg.2008.06.001 18565486 PMC2443840

[B86] Marx M. Haas C. A. Häussler U. (2013). Differential vulnerability of interneurons in the epileptic hippocampus. *Front. Cell. Neurosci.* 7:167. 10.3389/fncel.2013.00167 24098270 PMC3787650

[B87] Mele M. Costa R. O. Duarte C. B. (2019). Alterations in GABAA-receptor trafficking and synaptic dysfunction in brain disorders. *Front. Cell Neurosci*. 13:77. 10.3389/fncel.2019.00077 30899215 PMC6416223

[B88] Miri M. L. Vinck M. Pant R. Cardin J. A. (2018). Altered hippocampal interneuron activity precedes ictal onset. *eLife* 7:e40750. 10.7554/eLife.40750 30387711 PMC6245730

[B89] Moss S. J. Jacob T. C. Jurd R. (2008). GABA A receptor trafficking and its role in the dynamic modulation of neuronal inhibition. *Nat. Rev. Neurosci*. 9 331–343. 10.1038/nrn2370 18382465 PMC2709246

[B90] Muir J. Arancibia-Carcamo I. L. MacAskill A. F. Smith K. R. Griffin L. D. Kittler J. T. (2010). NMDA receptors regulate GABAA receptor lateral mobility and clustering at inhibitory synapses through serine 327 on the γ2 subunit. *Proc. Natl. Acad. Sci. U. S. A.* 107 16679–16684. 10.1073/pnas.1000589107 20823221 PMC2944765

[B91] Mukherjee J. Cardarelli R. A. Cantaut-Belarif Y. Deeb T. Z. Srivastava D. P. Tyagarajan S. K. (2017). Estradiol modulates the efficacy of synaptic inhibition by decreasing the dwell time of GABAA receptors at inhibitory synapses. *Proc. Natl. Acad. Sci. U. S. A.* 114 11763–11768. 10.1073/pnas.1705075114 29078280 PMC5676881

[B92] Mukherjee J. Kretschmannova K. Gouzer G. Maric H.-M. Ramsden S. Tretter V. (2011). The residence time of GABAARs at inhibitory synapses is determined by direct binding of the receptor α1 subunit to gephyrin. *J. Neurosci.* 31 14677–14687. 10.1523/JNEUROSCI.2001-11.2011 21994384 PMC3202462

[B93] Nakamura Y. Morrow D. H. Nathanson A. J. Henley J. M. Wilkinson K. A. Moss S. J. (2020). Phosphorylation on ser-359 of the α2 subunit in GABA type A receptors down-regulates their density at inhibitory synapses. *J. Biol. Chem.* 295 12330–12342. 10.1074/jbc.RA120.014303 32620552 PMC7458806

[B94] Nathanson A. J. Zhang Y. Smalley J. L. Ollerhead T. A. Santos M. A. R. Andrews P. M. (2019). Identification of a core amino acid motif within the α subunit of GABAARs that promotes inhibitory synaptogenesis and resilience to seizures. *Cell Rep.* 28 670–681.e8. 10.1016/j.celrep.2019.06.014 31315046 PMC8283774

[B95] Nicholson M. W. Sweeney A. Pekle E. Alam S. Ali A. B. Duchen M. (2018). Diazepam-induced loss of inhibitory synapses mediated by PLCδ/Ca2+/calcineurin signalling downstream of GABAA receptors. *Mol. Psychiatry* 23 1851–1867. 10.1038/s41380-018-0100-y 29904150 PMC6232101

[B96] Nicolas C. S. Amici M. Bortolotto Z. A. Doherty A. Csaba Z. Fafouri A. (2013). The role of JAK-STAT signaling within the CNS. *JAKSTAT* 2:e22925. 10.4161/jkst.22925 24058789 PMC3670265

[B97] Nishimura T. Schwarzer C. Gasser E. Kato N. Vezzani A. Sperk G. (2005). Altered expression of GABAA and GABAB receptor subunit mRNAs in the hippocampus after kindling and electrically induced status epilepticus. *Neuroscience* 134 691–704. 10.1016/j.neuroscience.2005.04.013 15951123

[B98] Nusser Z. Sieghart W. Benke D. Fritschy J. M. Somogyi P. (1996). Differential synaptic localization of two major gamma-aminobutyric acid type A receptor alpha subunits on hippocampal pyramidal cells. *Proc. Natl. Acad. Sci. U. S. A*. 93 11939–11944. 10.1073/pnas.93.21.11939 8876241 PMC38162

[B99] Okada M. Onodera K. Van Renterghem C. Sieghart W. Takahashi T. (2000). Functional correlation of GABA(A) receptor alpha subunits expression with the properties of IPSCs in the developing thalamus. *J. Neurosci*. 20 2202–2208. 10.1523/JNEUROSCI.20-06-02202.2000 10704495 PMC6772493

[B100] Owen B. Bichler E. Benveniste M. (2021). Excitatory synaptic transmission in hippocampal area CA1 is enhanced then reduced as chronic epilepsy progresses. *Neurobiol. Dis.* 154:105343. 10.1016/j.nbd.2021.105343 PMC8113127 33753293

[B101] Papadopoulos T. Eulenburg V. Reddy-Alla S. Mansuy I. M. Li Y. Betz H. (2008). Collybistin is required for both the formation and maintenance of GABAergic postsynapses in the hippocampus. *Mol. Cell Neurosci.* 39 161–169. 10.1016/j.mcn.2008.06.006 18625319

[B102] Park J. Choi Y. Jung E. Lee S. Sohn J. Chung W. (2021). Microglial MERTK eliminates phosphatidylserine-displaying inhibitory post-synapses. *EMBO J*. 40:e107121. 10.15252/embj.2020107121 PMC8327958 34013588

[B103] Peng Z. Huang C. S. Stell B. M. Mody I. Houser C. R. (2004). Altered expression of the delta subunit of the GABAA receptor in a mouse model of temporal lobe epilepsy. *J. Neurosci*. 24 8629–8639. 10.1523/JNEUROSCI.2877-04.2004 15456836 PMC6729896

[B104] Petrini E. M. Ravasenga T. Hausrat T. J. Iurilli G. Olcese U. Racine V. (2014). Synaptic recruitment of gephyrin regulates surface GABAA receptor dynamics for the expression of inhibitory LTP. *Nat. Commun.* 5:3921. 10.1038/ncomms4921 PMC4059940 24894704

[B105] Pfisterer U. Petukhov V. Demharter S. Meichsner J. Thompson J. J. Batiuk M. Y. (2020). Identification of epilepsy-associated neuronal subtypes and gene expression underlying epileptogenesis. *Nat. Commun*. 11:5988. 10.1038/s41467-020-18752-7 PMC7678822 33214565

[B106] Pirker S. Schwarzer C. Czech T. Baumgartner C. Pockberger H. Maier H. (2003). Increased expression of GABAA receptor β-subunits in the hippocampus of patients with temporal lobe epilepsy. *J. Neuropathol. Exp. Neurol.* 62 820–834. 14503638 10.1093/jnen/62.8.820

[B107] Poulopoulos A. Aramuni G. Meyer G. Soykan T. Hoon M. Papadopoulos T. (2009). Neuroligin 2 drives postsynaptic assembly at perisomatic inhibitory synapses through gephyrin and collybistin. *Neuron* 63 628–642. 10.1016/j.neuron.2009.08.023 19755106

[B108] Pribiag H. Stellwagen D. (2013). TNF-α downregulates inhibitory neurotransmission through protein phosphatase 1-dependent trafficking of GABAA receptors. *J. Neurosci.* 33 15879–15893. 10.1523/JNEUROSCI.0530-13.2013 24089494 PMC6618471

[B109] Rabow L. E. Russek S. J. Farb D. H. (1995). From ion currents to genomic analysis: Recent advances in GABAA receptor research. *Synapse* 21 189–274. 10.1002/syn.890210302 8578436

[B110] Ramsdell J. S. (2010). Neurological disease rises from ocean to bring model for human epilepsy to life. *Toxins* 2 1646–1675. 10.3390/toxins2071646 22069654 PMC3153267

[B111] Raol Y. H. Lund I. V. Bandyopadhyay S. Zhang G. Roberts D. S. Wolfe J. H. (2006). Enhancing GABA(A) receptor alpha 1 subunit levels in hippocampal dentate gyrus inhibits epilepsy development in an animal model of temporal lobe epilepsy. *J. Neurosci*. 26 11342–11346. 10.1523/JNEUROSCI.3329-06.2006 17079662 PMC6674546

[B112] Roberts D. S. Hu Y. Lund I. V. Brooks-Kayal A. R. Russek S. J. (2006). Brain-derived neurotrophic factor (BDNF)-induced synthesis of early growth response factor 3 (Egr3) controls the levels of type A GABA receptor alpha 4 subunits in hippocampal neurons. *J. Biol. Chem*. 281 29431–29435. 10.1074/jbc.C600167200 16901909

[B113] Roberts D. S. Raol Y. H. Bandyopadhyay S. Lund I. V. Budreck E. C. Passini M. A. (2005). Egr3 stimulation of GABRA4 promoter activity as a mechanism for seizure-induced up-regulation of GABA(A) receptor alpha4 subunit expression. *Proc. Natl. Acad. Sci. U. S. A*. 102 11894–11899. 10.1073/pnas.0501434102 16091474 PMC1187961

[B114] Roca D. J. Rozenberg I. Farrant M. Farb D. H. (1990). Chronic agonist exposure induces down-regulation and allosteric uncoupling of the gamma-aminobutyric acid/benzodiazepine receptor complex. *Mol. Pharmacol*. 37 37–43. 2153908

[B115] Rogawski M. A. Löscher W. Rho J. M. (2016). Mechanisms of action of antiseizure drugs and the ketogenic diet. *Cold Spring Harb. Perspect. Med.* 6:a022780. 10.1101/cshperspect.a022780 26801895 PMC4852797

[B116] Roussarie J. Yao V. Rodriguez-Rodriguez P. Oughtred R. Rust J. Plautz Z. (2020). Selective neuronal vulnerability in alzheimer’s disease: A network-based analysis. *Neuron* 107 821–835.e12. 10.1016/j.neuron.2020.06.010 32603655 PMC7580783

[B117] Russek S. J. Bandyopadhyay S. Farb D. H. (2000). An initiator element mediates autologous downregulation of the human type A γ-aminobutyric acid receptor β1 subunit gene. *Proc. Natl. Acad. Sci. U. S. A*. 97 8600–8605. 10.1073/pnas.97.15.8600 10900018 PMC26994

[B118] Saha S. Hu Y. Martin S. C. Bandyopadhyay S. Russek S. J. Farb D. H. (2013). Polycomblike protein PHF1b: A transcriptional sensor for GABA receptor activity. *BMC Pharmacol. Toxicol*. 14:37. 10.1186/2050-6511-14-37 23879974 PMC3734045

[B119] Sayin U. Osting S. Hagen J. Rutecki P. Sutula T. (2003). Spontaneous seizures and loss of axo-axonic and axo-somatic inhibition induced by repeated brief seizures in kindled rats. *J. Neurosci*. 23 2759–2768. 10.1523/JNEUROSCI.23-07-02759.2003 12684462 PMC6742074

[B120] Schofield C. M. Huguenard J. R. (2007). GABA affinity shapes IPSCs in thalamic nuclei. *J. Neurosci*. 27 7954–7962. 10.1523/JNEUROSCI.0377-07.2007 17652586 PMC6672741

[B121] Schwarzer C. Tsunashima K. Wanzenböck C. Fuchs K. Sieghart W. Sperk G. (1997). GABAA receptor subunits in the rat hippocampus II: Altered distribution in kainic acid-induced temporal lobe epilepsy. *Neuroscience* 80 1001–1017. 10.1016/s0306-4522(97)00145-0 9284056

[B122] Serratto G. M. Pizzi E. Murru L. Mazzoleni S. Pelucchi S. Marcello E. (2020). The epilepsy-related protein PCDH19 regulates tonic inhibition GABAAR kinetics, and the intrinsic excitability of hippocampal neurons. *Mol. Neurobiol*. 57 5336–5351. 10.1007/s12035-020-02099-7 32880860 PMC7541378

[B123] Shao L. Habela C. W. Stafstrom C. E. (2019). Pediatric epilepsy mechanisms: Expanding the paradigm of excitation/inhibition imbalance. *Children* 6:23. 10.3390/children6020023 30764523 PMC6406372

[B124] Shimojima K. Sugawara M. Shichiji M. Mukaida S. Takayama R. Imai K. (2011). Loss-of-function mutation of collybistin is responsible for X-linked mental retardation associated with epilepsy. *J. Hum. Genet.* 56 561–565. 10.1038/jhg.2011.58 21633362

[B125] Shin S. M. Skaar S. Danielson E. Lee S. H. (2020). Aberrant expression of S-SCAM causes the loss of GABAergic synapses in hippocampal neurons. *Sci. Rep.* 10:83. 10.1038/s41598-019-57053-y 31919468 PMC6952429

[B126] Shumate M. D. Lin D. D. Gibbs I. I. I. J. W. Holloway K. L. Coulter D. A. (1998). GABAA receptor function in epileptic human dentate granule cells: Comparison to epileptic and control rat. *Epilepsy Res*. 32 114–128. 10.1016/s0920-1211(98)00045-x 9761314

[B127] Sills G. J. Rogawski M. A. (2020). Mechanisms of action of currently used antiseizure drugs. *Neuropharmacology* 168:107966. 10.1016/j.neuropharm.2020.107966 32120063

[B128] Smith L. Singhal N. Achkar C. M. E. Truglio G. Beth R.-S. Sullivan J. (2018). PCDH19-related epilepsy is associated with a broad neurodevelopmental spectrum. *Epilepsia* 59 679–689. 10.1111/epi.14003 29377098 PMC6264912

[B129] Sperk G. (1994). Kainic acid seizures in the rat. *Progr. Neurobiol*. 42 1–32. 10.1016/0301-0082(94)90019-1 7480784

[B130] Sperk G. Pirker S. Gasser E. Wieselthaler A. Bukovac A. Kuckukhidze G. (2021). Increased expression of GABAA receptor subunits associated with tonic inhibition in patients with temporal lobe epilepsy. *Brain Commun.* 3:fcab239. 10.1093/braincomms/fcab239 34708207 PMC8545616

[B131] Stefanits H. Milenkovic I. Mahr N. Pataraia E. Baumgartner C. Hainfellner J. (2019). Alterations in GABAA receptor subunit expression in the amygdala and entorhinal cortex in human temporal lobe epilepsy. *J. Neuropathol. Exp. Neurol*. 78 1022–1048. 10.1093/jnen/nlz085 31631219

[B132] Sun C. Sieghart W. Kapur J. (2004). Distribution of α1, α4, γ2, and δ subunits of GABAA receptors in hippocampal granule cells. *Brain Res*. 1029 207–216. 10.1016/j.brainres.2004.09.056 15542076 PMC2892719

[B133] Tanaka T. Saito H. Matsuki N. (1997). Inhibition of GABAA synaptic responses by brain-derived neurotrophic factor (BDNF) in rat hippocampus. *J. Neurosci*. 17 2959–2966. 10.1523/JNEUROSCI.17-09-02959.1997 9096132 PMC6573653

[B134] Téllez-Zenteno J. F. Hernández-Ronquillo L. (2012). A review of the epidemiology of temporal lobe epilepsy. *Epilepsy Res. Treat*. 2012:630853. 10.1155/2012/630853 22957234 PMC3420432

[B135] Terunuma M. Xu J. Vithlani M. Sieghart W. Kittler J. Pangalos M. (2008). Deficits in phosphorylation of GABA(A) receptors by intimately associated protein kinase C activity underlie compromised synaptic inhibition during status epilepticus. *J. Neurosci*. 28 376–384. 10.1523/JNEUROSCI.4346-07.2008 18184780 PMC2917223

[B136] Thomas P. Mortensen M. Hosie A. M. Smart T. G. (2005). Dynamic mobility of functional GABAA receptors at inhibitory synapses. *Nat. Neurosci.* 8 889–897. 10.1038/nn1483 15951809

[B137] Tóth K. Maglóczky Z. (2014). The vulnerability of calretinin-containing hippocampal interneurons to temporal lobe epilepsy. *Front. Neuroanat.* 8:100. 10.3389/fnana.2014.00100 25324731 PMC4179514

[B138] Tsunashima K. Schwarzer C. Kirchmair E. Sieghart W. Sperk G. (1997). GABAA receptor subunits in the rat hippocampus III: Altered messenger RNA expression in kainic acid-induced epilepsy. *Neuroscience* 80 1019–1032. 10.1016/s0306-4522(97)00144-9 9284057

[B139] Tyagarajan S. K. Ghosh H. Yévenes G. E. Imanishi S. Y. Zeilhofer H. U. Gerritis B. (2013). Extracellular signal-regulated kinase and glycogen synthase kinase 3β regulate gephyrin postsynaptic aggregation and GABAergic synaptic function in a calpain-dependent mechanism. *J. Biol. Chem*. 288 9634–9647. 10.1074/jbc.M112.442616 23408424 PMC3617267

[B140] Tyagarajan S. K. Ghosh H. Yévenes G. E. Nikonenko I. Eeling C. Schwerdel C. (2011). Regulation of GABAergic synapse formation and plasticity by GSK3β-dependent phosphorylation of gephyrin. *Proc. Natl. Acad. Sci. U. S. A.* 108 379–384. 10.1073/pnas.1011824108 21173228 PMC3017200

[B141] Uchino K. Kawano H. Tanaka Y. Adanniya Y. Asahara A. Deshimaru M. (2021). Inhibitory synaptic transmission is impaired at higher extracellular Ca2+ concentrations in Scn1a+/- mouse model of dravet syndrome. *Sci. Rep*. 11:10634. 10.1038/s41598-021-90224-4 34017040 PMC8137694

[B142] Varoqueaux F. Aramuni G. Rawson R. L. Mohrmann R. Missler M. Gottmann K. (2006). Neuroligins determine synapse maturation and function. *Neuron* 51 741–754. 10.1016/j.neuron.2006.09.003 16982420

[B143] Varoqueaux F. Jamain S. Brose N. (2004). Neuroligin 2 is exclusively localized to inhibitory synapses. *Eur. J. Cell Biol*. 83 449–456. 10.1078/0171-9335-00410 15540461

[B144] Vaz S. H. Cristóvão-Ferreira S. Ribeiro J. A. Sebastião A. M. (2008). Brain-derived neurotrophic factor inhibits GABA uptake by the rat hippocampal nerve terminals. *Brain Res*. 1219 19–25. 10.1016/j.brainres.2008.04.008 18539266

[B145] Vithlani M. Terunuma M. Moss S. J. (2011). The dynamic modulation of GABAA receptor trafficking and its role in regulating the plasticity of inhibitory synapses. *Physiol. Rev*. 91 1009–1022. 10.1152/physrev.00015.2010 21742794 PMC4382539

[B146] Waloschková E. Gonzalez-Ramos A. Mikroulis A. Kudláèek J. Andersson M. Ledri M. (2021). Human stem cell-derived GABAergic interneurons establish efferent synapses onto host neurons in rat epileptic hippocampus and inhibit spontaneous recurrent seizures. *Int. J. Mol. Sci.* 22:13243. 10.3390/ijms222413243 34948040 PMC8705828

[B147] Weston M. C. Chen H. Swann J. W. (2014). Loss of mTOR repressors Tsc1 or pten has divergent effects on excitatory and inhibitory synaptic transmission in single hippocampal neuron cultures. *Front. Mol. Neurosci*. 7:1. 10.3389/fnmol.2014.00001 24574959 PMC3922082

[B148] Wu K. Castellano D. Tian Q. Lu W. (2021a). Distinct regulation of tonic GABAergic inhibition by NMDA receptor subtypes. *Cell Rep.* 37:109960. 10.1016/j.celrep.2021.109960 34758303 PMC8630577

[B149] Wu K. Han W. Tian Q. Li Y. Lu W. (2021b). Activity- and sleep-dependent regulation of tonic inhibition by Shisa7. *Cell Rep*. 34:108899. 10.1016/j.celrep.2021.108899 33761345 PMC8025742

[B150] Wu M. Tian H. Liu X. Lai J. H. C. Du S. Xia J. (2018). Impairment of inhibitory synapse formation and motor behavior in mice lacking the NL2 binding partner LHFPL4/GARLH4. *Cell Rep*. 23 1691–1705. 10.1016/j.celrep.2018.04.015 29742426

[B151] Wu T. Chen S. Brinton R. D. (2011). Membrane estrogen receptors mediate calcium signaling and MAP kinase activation in individual hippocampal neurons. *Brain Res.* 1379 34–43. 10.1016/j.brainres.2011.01.034 21241678 PMC3050738

[B152] Yamasaki T. Hoyos-Ramirez E. Martenson J. S. Morimoto-Tomita M. Tomita S. (2017). GARLH family proteins stabilize GABAA receptors at synapses. *Neuron* 93 1138–1152.e6. 10.1016/j.neuron.2017.02.023 28279354 PMC5347473

[B153] Zhang B. Chen L. Y. Liu X. Maxeiner S. Lee S.-J. Gokce O. (2015). Neuroligins sculpt cerebellar purkinje-cell circuits by differential control of distinct classes of synapses. *Neuron* 87 781–796. 10.1016/j.neuron.2015.07.020 26291161 PMC4545494

[B154] Zhang N. Wei W. Mody I. Houser C. R. (2007). Altered localization of GABAA receptor subunits on dentate granule cell dendrites influences tonic and phasic inhibition in a mouse model of epilepsy. *J. Neurosci*. 27 7520–7531. 10.1523/JNEUROSCI.1555-07.2007 17626213 PMC6672608

[B155] Zhang T. Yu F. Xu H. Chen M. Chen X. Guo L. (2021). Dysregulation of REV-ERBα impairs GABAergic function and promotes epileptic seizures in preclinical models. *Nat. Commun*. 12:1216. 10.1038/s41467-021-21477-w.107 PMC7900242 33619249

